# On Markovianity and classicality in multilevel spin–boson models

**DOI:** 10.1038/s41598-023-28606-z

**Published:** 2023-01-27

**Authors:** Dariusz Chruściński, Samaneh Hesabi, Davide Lonigro

**Affiliations:** 1 Institute of Physics, Faculty of Physics, Astronomy and Informatics, Nicolaus Copernicus University, Grudziadzka 5/7, 87-100 Toruń, Poland; 2 Dipartimento di Fisica and MECENAS, Università di Bari, 70126 Bari, Italy; 3 INFN, Sezione di Bari, 70126 Bari, Italy

**Keywords:** Mathematics and computing, Physics

## Abstract

We provide a detailed discussion about the unitary and reduced evolution induced by family of Hamiltonian models describing a multilevel system, with a ground state and a possibly multilevel excited sector, coupled to a multimode boson field via a rotating-wave interaction. We prove explicitly that the system, in the limit in which the coupling is flat with respect to the boson frequencies, is Markovian under sharp measurements in arbitrary bases; we also find necessary and sufficient conditions under which the process is classical, i.e. its family of multitime joint probability distributions satisfies the Kolmogorov consistency condition, and may thus be equivalently obtained by a classical stochastic process.

## Introduction

Long before the dawn of quantum mechanics, randomness has always occupied a central role in the description of natural phenomena. Since the publication of A. Kolmogorov’s pioneering book^1^, the modern theory of stochastic processes has been developed in a measure-theoretical axiomatic framework which proves to be powerful and flexible enough for the description of diverse classical physical phenomena—Brownian motion being a paradigmatic example—as well as having applications in other natural and social sciences^2^.

In this framework, a (classical) stochastic process corresponds to a collection of functions $\{X(t)\}_{t\in T}$, with $T\subset {\mathbb {R}}$ being the set of times, from a suitably chosen probability space to some set of values $\mathcal {X}$. Most importantly, it is associated with a family of *joint probability distributions* $\{{\mathbb {P}}_n\}_{n\in {\mathbb {N}}}$ defined as follows: given a family of values $x_1,\ldots ,x_n\in {\mathcal {X}}$ and a set of times $t_1,\ldots ,t_n$, the quantity

1 $$\begin{aligned} {\mathbb {P}}_n(x_n,t_n;x_{n-1},t_{n-1};\ldots ;x_1,t_1)=\textrm{Prob}\left\{ X(t_1)=x_1,\ldots ,X(t_n)=x_n\right\} \end{aligned}$$

represents the probability that the system, probed at each time $t_j$, is measured in the state $x_j$. Interestingly, the distributions associated with a stochastic process automatically satisfy a *consistency condition* which, for a discrete set of values, reads as follows:2that is, for all $k<n$ all *k*-time joint distributions can be obtained via marginalization from the *n*-time one. Conversely, the celebrated Kolmogorov extension theorem guarantees that every *consistent* (i.e. satisfying Eq. 2) family of joint probability distributions can be obtained from a classical stochastic process. Importantly, as pointed out in^3,4^, Eq. (2) essentially means that not performing a measurement at the time $t_j$ is operationally indistinguishable from performing the measurement at the time $t_j$ and then “forgetting” about the outcome, i.e. averaging over all possible outcomes—that is, *measurements do not alter the state of the system*.

Markovian processes are particularly important. A stochastic process is said to be *Markovian* whenever the following property holds for all $x_1,\ldots ,x_n\in {\mathcal {X}}$ and $t_1\le \ldots \le t_n\in T$:

3 $$\begin{aligned} {\mathbb {P}}(x_n,t_n|x_{n-1},t_{n-1};\dots ;x_1,t_1)={\mathbb {P}}(x_n,t_n|x_{n-1},t_{n-1}), \end{aligned}$$

with ${\mathbb {P}}(\cdot |\cdot )$ being the conditional probability; in words, the process is Markovian if the information contained in the most recent measure fully determines the behavior of the system at all future times, regardless the outcome of earlier measurements. While this definition is apparently asymmetric in time, equivalent definitions of Markovianity which restore the symmetry between “future” and “past” can be given; see e.g.^5^, Definition 7.1. Essentially, Markovianity means that the past and the future are conditionally mutually independent with respect to the present time. In this sense, a Markovian process is memoryless. Most importantly, as a straightforward consequence of Eq. (3), the whole family of joint probabilities can be entirely reconstructed from the single-time probability ${\mathbb {P}}_1(x,t)$ and the transition probability ${\mathbb {P}}(x,t|y,s)$ via

4 $$\begin{aligned} {\mathbb {P}}_n(x_n,t_n;x_{n-1},t_{n-1};\ldots ;x_1,t_1)={\mathbb {P}}(x_n,t_n|x_{n-1},t_{n-1})\cdots {\mathbb {P}}(x_2,t_2|x_{1},t_{1}){\mathbb {P}}_1(x_1,t_1); \end{aligned}$$

besides, by Eqs. (3)–(4), the whole family of consistency conditions (2) reduces to the Chapman–Kolmogorov equations^2^:

5 $$\begin{aligned} {\mathbb {P}}_1(x,t)= & {} \sum _{z\in {\mathcal {X}}}{\mathbb {P}}(x,t|z,r){\mathbb {P}}_1(z,r),\qquad \quad \,\, t\ge r \end{aligned}$$

6 $$\begin{aligned} {\mathbb {P}}(x,t|y,s)= & {} \sum _{z\in {\mathcal {X}}}{\mathbb {P}}(x,t|z,r){\mathbb {P}}(z,r|y,s),\qquad t\ge r\ge s. \end{aligned}$$

Quantum mechanics is, in its essence, a probabilistic theory: the outcome of any quantum mechanical experiment is famously non-deterministic. However, this is far from being the only novelty of quantum mechanics: the plethora of new phenomena unveiled by a close scrutiny to the quantum world, like entanglement and decoherence, makes the language of classical stochastic processes unsuited to quantum mechanics. To put it in mathematical terms, let us consider an open quantum system described by a Hamiltonian ${\textbf {H}}$ on a Hilbert space $\mathcal {H}=\mathcal {H}_{\textrm S}\otimes \mathcal {H}_{{\textrm B}}$, with $\mathcal {H}_{{\textrm{S}}}$ being the space associated with the experimentally accessible system and $\mathcal {H}_{{\textrm{B}}}$ with the external environment (or bath). Suppose that said system is initially prepared at a time $t_0$ in a state associated with some density operator $\varrho _0\in {\mathcal {B}}(\mathcal {H})$, and repeatedly probed at times $t_n\ge t_{n-1}\ge \dots t_1\ge t_0$ via some family of sharp projective measurements in an orthonormal basis $\{|x\rangle \}_{x\in {\mathcal {X}}}$ of $\mathcal {H}_{{\textrm{S}}}$. The laws of quantum mechanics provide us a simple and elegant rule to construct the joint probability distribution associated with any such sequence of measurements:

7 $$\begin{aligned} {\mathbb {P}}_n(x_n,t_n;\dots ;x_1,t_1)={\textrm{Tr}}\left[ \left( {\mathcal {P}}_{x_n}\otimes {\mathcal {I}}_{{\textrm{B}}}\right) {\mathcal {U}}_{t_n-t_{n-1}}\cdots \left( {\mathcal {P}}_{x_1}\otimes {\mathcal {I}}_{{\textrm{B}}}\right) {\mathcal {U}}_{t_1-t_0}(\varrho _{0})\right] , \end{aligned}$$

with ${\mathcal {U}}_t={\textrm{e}}^{-{\textrm i}t{\textbf {H}}}(\cdot ){\textrm{e}}^{{\textrm i}t{\textbf {H}}}$ representing the free evolution of the system+environment induced by ${\textbf {H}}$ on $\mathcal {H}_{{\textrm{S}}}\otimes \mathcal {H}_{{\textrm{B}}}$, ${\mathcal {I}}_{{\textrm{B}}}$ the identity map on the environment space $\mathcal {H}_{{\textrm{B}}}$, and ${\mathcal {P}}_x = P_x (\cdot )P_x$, with $P_x = |x\rangle \!\langle x|$. Eq. (7) defines a legitimate family of joint probability distributions; however, in general they *do not satisfy* the consistency condition (2), and thus are not associated with any underlying classical stochastic process. This is ultimately related to the fact that, in general, measurement *do* disturb the state of a quantum system—not performing a measurement is not the same as performing said measurement and forgetting it, in contrast with Eq. (2).

Inspired by the theory of stochastic processes, and following the same point of view as in^3,4^, we shall adopt hereafter the following definitions. Given an open quantum system associated with a total Hamiltonian ${\textbf {H}}$ on $\mathcal {H}=\mathcal {H}_{{\textrm{S}}}\otimes \mathcal {H}_{{\textrm{B}}}$, initially prepared in some collective state $\varrho _0$, and a fixed orthonormal basis of $\mathcal {H}_{{\textrm{B}}}$, we shall denote as *quantum process* any sequence of sharp measurements in said basis on the system; defining the corresponding family of joint probability distributions as in Eq. (7), the process is said to be

- *Markovian*, if the Markov property (3) holds.
- *classical*, if the consistency condition (2) (or, in the Markov case, Eq. (6)) holds.

Importantly, both definitions are strictly *dependent* on the choice of the measurement basis.

In this regard, it is worth recalling that no universally accepted definition of quantum Markovianity exists. Diverse mathematical properties, each focusing on particular aspects of the problem under investigation, have been—more or less formally—put forward as definitions of quantum Markovianity. Some of them solely involve the properties of the reduced dynamics: for instance, the completely positive divisibility^6^ (and, as a particular case, the semigroup property) and the monotonicity of the distinguishability between arbitrary states^7^. Other approaches take into account the full unitary dynamics of the system *and* the environment in order to account for the unraveling of information backflow via external interventions or measurements on the system: among many others, the validity of the factorization (or Born) approximation—roughly speaking, the idea that the environment, not “feeling” the action of the system, evolves independently of the presence of the latter—or the quantum regression formula. An exhaustive discussion of all concepts of Markovianity in quantum mechanics and the hierarchical relations between them is reported in^8^. One should always take into account this ambiguity when dealing with concepts of (non–)Markovianity in open quantum systems (cf. the recent reviews^9–12^). The approach of^3,4^ adopted in this paper is closely related to the mathematical formulation of quantum Markov stochastic processes proposed in^13–15^ (cf. also the recent review^16^). Moreover, it is closely related to the recent approach to quantum Markovianity proposed in^17–19^ in which the Markovianity of the corresponding process is characterized in terms of the so-called quantum process tensor of the system. The factorization of the process tensor is essentially equivalent to the validity of quantum regression^8^. For a discussion of Markovianity based on the quantum regression formula see also^20,21^.

Adopting the above definition of Markovianity^3,4^, a useful characterization of classicality was first provided in^3^ for dynamical semigroups, and then extended in^4^ to general quantum Markov processes. Classicality was shown to be crucially interrelated with the generation of coherence: fundamentally, a Markovian multitime statistics fails to be classical if and only if the dynamics generates coherences and subsequently turns them into populations—mathematically, classicality holds whenever the process can be represented by means of non-coherence-generating-and-detecting (NCGD) maps. Furthermore, in^4^ a similar characterization was obtained for (possibly) non-Markovian processes: while in this case the absence of coherence does not guarantee classicality, they managed to provide a direct connection between classicality and the vanishing of quantum discord between the evolving system and its environment.

Inspired by such general results, the scope of the present paper is to provide an explicit discussion of Markovianity and classicality in the context of a specific, paradigmatic class of open quantum systems: spin–boson models and their generalizations. The interest in such models is not recent. Since decades, spin–boson models and their generalizations have been claiming a primary role in the theory of open quantum systems^22–26^, finding applications as toy models for the description of several quantum phenomena like noise and decoherence^27–32^ as well as practical applications^22,25,27–29,33,34^. The Markovianity of the evolution induced by spin–boson models, in many of the possible declensions listed above, has also been analyzed by several authors^9–11,22,25^; spin–boson models also provide simple examples in which, via a suitable choice of the form factor, the onset of non-Markovianity can be delayed at arbitrarily large times^35,36^.

In this regard, we shall consider a class of models describing a quantum system (e.g. atom) with a single ground energy level and *d* excited energy levels, interacting with a structured boson bath, first introduced in Ref.^37^, and further studied in^38^. Such models belong to the class of generalized spin–boson models^39–43^, their self-adjointness being ensured whenever all form factors modulating the system–bath interaction are normalizable (square-integrable)^39^ but also for suitable classes of non-normalizable form factors^44^. For such models, after recalling the main properties of the unitary evolution in the single-excitation sector, we shall provide an explicit, constructive discussion of Markovianity and classicality in the limit in which all form factors are flat—that is, all boson frequencies, positive and negative, are coupled to the atom with the same strength. Precisely, in such a limit the model will be proven to be Markovian—in the sense of Eq. (3)—with respect to measurements in an *arbitrary* orthogonal basis. Furthermore, necessary and sufficient conditions for its classicality in this limit will be found, and discussed at the light of the existing general results in^3,4^ about classicality in quantum processes.

The paper is structured as follows. After recalling in Sect. "Preliminary: Markovianity and quantum regression" the link between quantum Markovianity and regression, in Sect. "Generalities on the multilevel spin–boson model" we introduce the class of multilevel spin–boson models under study, revise the exact computation of the dynamics of any single-excited state, derive the corresponding reduced dynamics on the system, and study its divisibility properties. In Sect. "The flat coupling limit" we take into account the limiting case of flat atom-field couplings, studying the corresponding properties of the reduced dynamics. Finally, Sect. "Markovianity and classicality" is devoted to the main results of the paper: proving that the model is indeed Markovian, in the sense discussed above, with respect to arbitrary sharp measurements, and proving simple conditions under which it is (non–)classical. Some concluding remarks are outlined in Sect. "Conclusions".

## Preliminary: Markovianity and quantum regression

For our purposes, it will be useful to briefly discuss the link between Markovianity and the validity of the general quantum regression formula (GQRF)^8,22,45^. Consider a open quantum system corresponding to a self-adjoint Hamiltonian ${\textbf {H}}$ on a bipartite space $\mathcal {H}=\mathcal {H}_{{\textrm{S}}}\otimes \mathcal {H}_{{\textrm{B}}}$, with $\dim \mathcal {H}_{{\textrm{S}}}=d+1$, giving rise to a unitary evolution ${\mathcal {U}}_t$ of the “system + bath”, and a fixed initial state $\rho _{{\textrm{B}}}$ of the bath; suppose that the reduced evolution of the system, associated with the family of propagators $\Lambda _{t,s}(\rho ) = {\textrm{Tr}}_{{\textrm{B}}}\,{\mathcal {U}}_{t-s}(\rho \otimes \rho _{{\textrm{B}}})$, is CP-divisible. We say that a pair $({\mathcal {U}}_t,\rho _{{\textrm{B}}})$ satisfies the GQRF if, for any collection of times $t_n> t_{n-1}>\cdots> t_1 > t_0$ and two sets of system operators $\{X_0,X_1,\ldots ,X_n\}$ and $\{Y_0,Y_1,\ldots ,Y_n\}$, one has the following relation between multitime correlation functions:

8 $$\begin{aligned} {\textrm{Tr}}[\tilde{{\mathcal {E}}}_n {\mathcal {U}}_{t_n-t_{n-1}} \cdots \tilde{{\mathcal {E}}}_1\,{\mathcal {U}}_{t_1-t_0}(\rho \otimes \rho _{{\textrm{B}}})] = {\textrm{Tr}}[{\mathcal {E}}_n \Lambda _{t_n,t_{n-1}} \cdots {\mathcal {E}}_1 \Lambda _{t_1,t_0}(\rho )] , \end{aligned}$$

where ${\mathcal {E}}_k=X_k\cdot Y_k$ and $\tilde{{\mathcal {E}}}_k={\mathcal {E}}_k\otimes {\mathcal {I}}_{{\textrm{B}}}$. Equation (8) means that all correlation functions for the “system+bath” evolution can be computed in terms of the dynamical map of the system alone. In particular, in such a case, considering projective measurements w.r.t. an orthonormal basis $\{|x\rangle \}_{x=0,\ldots ,d}$ in the Hilbert space ${\mathcal {H}}_{{\textrm{S}}}$, and setting

9 $$\begin{aligned} P_x=| x \rangle \!\langle x | ,\qquad {\mathcal {P}}_x=P_x(\cdot )P_x, \end{aligned}$$

we get

10 $$\begin{aligned} {\mathbb {P}}_n(x_n,t_n;\dots ;x_1,t_1)= & {} {\textrm{Tr}}\left[ \left( {\mathcal {P}}_{x_n}\otimes {\mathcal {I}}_{{\textrm{B}}}\right) {\mathcal {U}}_{t_n-t_{n-1}}\cdots \left( {\mathcal {P}}_{x_1}\otimes {\mathcal {I}}_{{\textrm{B}}}\right) {\mathcal {U}}_{t_1-t_0}(\rho \otimes \rho _{{\textrm{B}}})\right] \nonumber \\= & {} {\textrm{Tr}}\left[ {\mathcal {P}}_{x_n}\Lambda _{t_n,t_{n-1}}\cdots {\mathcal {P}}_{x_1}\Lambda _{t_1-t_0}(\rho )\right] \nonumber \\= & {} \langle x_1|\Lambda _{t_1,t_0}(\rho )|x_1\rangle \prod _{j=1}^{n-1} \langle x_{j+1}| \Lambda _{t_{j+1},t_{j}} \left( | x_{j} \rangle \!\langle x_{j} | \right) |x_{j+1}\rangle , \end{aligned}$$

where the last equality is an identity which simply follows from the explicit definition of the maps ${\mathcal {P}}_x$. This observation is at the core of the relation between Markovianity and regression, which, while well-known, shall be recalled explicitly for the sake of completeness:

### Proposition 2.1

The following statements are equivalent:

(i) the regression equality (10) holds for all *n*;
(ii) the process is Markovian, and Eq. (10) holds for $n=1,2$;
(iii) the process is Markovian, and satisfies
  11 $$\begin{aligned} {\mathbb {P}}_1(x_1,t_1)= & {} \langle x_1|\Lambda _{t_1}(\rho )|x_1\rangle ; \end{aligned}$$
  12 $$\begin{aligned} {\mathbb {P}}(x_2,t_2|x_1,t_1)= & {} \langle x_2|\Lambda _{t_2,t_1}(| x_1 \rangle \!\langle x_1 | )|x_2\rangle . \end{aligned}$$

### Proof

The equivalence (ii)$\iff$(iii) is an immediate consequence of Eq. (10). (i)$\implies$(ii) is an immediate consequence of the fact that, if (i) holds, then the full family of joint probabilities is given by Eq. (10) and thus

13 $$\begin{aligned} {\mathbb {P}}(x_n,t_n|x_{n-1},t_{n-1};\ldots ;x_1,t_1)=\langle x_n|\Lambda _{t_n,t_{n-1}}\left( | x_{n-1} \rangle \!\langle x_{n-1} | \right) |x_n\rangle ={\mathbb {P}}(x_n,t_n|x_{n-1},t_{n-1}). \end{aligned}$$

Vice versa, suppose that (ii) is true. Since the process is Markovian, the family of joint probabilities can be entirely reconstructed as such:

14 $$\begin{aligned} {\mathbb {P}}_n(x_n,t_n;\ldots ;x_1,t_1)={\mathbb {P}}(x_n,t_n|x_{n-1},t_{n-1})\cdots {\mathbb {P}}(x_2,t_2|x_1,t_1){\mathbb {P}}_1(x_1,t_1). \end{aligned}$$

By Eq. (14) and the fact that Eq. (10) holds for $n=1,2$, one easily concludes that the latter equality holds for all *n*. $\square$

This shows the link between the validity of the regression formula *for a fixed orthonormal basis*, and the Markovianity in the same basis: *n*-point regression implies Markovianity, and Markovianity plus 1-point and 2-point regression implies *n*-point regression. We shall make use of this useful property later on.

## Generalities on the multilevel spin–boson model

We shall consider a $(d+1)$-level quantum system living in the Hilbert space $\mathcal {H}_{{\textrm{S}}}=\mathcal {H}_{{\textrm{e}}}\oplus \mathcal {H}_{{\textrm g}}$, with $\dim \mathcal {H}_{{\textrm{e}}}=d$ and $\dim \mathcal {H}_{{\textrm g}}=1$. $\mathcal {H}_{{\textrm{e}}}$ corresponds to a *d*-dimensional excited sector, whereas $\mathcal {H}_{{\textrm g}}$ is spanned by the ground state $|{\textrm 0}\rangle$. The system is coupled to a multimode boson bath, which (for simplicity) will be taken as a *d*-mode bath, with the total system-bath Hamiltonian given by ($\hbar =1$)

15 $$\begin{aligned} {\textbf{H}} = H_{\text{e}} \otimes \mathrm{I}_{\rm B} + \mathrm{I}_{{\textrm{S}}} \otimes \sum _{j=1}^d \int {\textrm d}\omega \;\omega \, b^\dagger _j(\omega ) b_j(\omega ) + H_{{\textrm int}} , \end{aligned}$$

where $H_{{\textrm{e}}}$ is the free Hamiltonian of the excited sector of the system, and the interaction term reads

16 $$\begin{aligned} H_{{\textrm int}} = \sum _{j=1}^d\int {\textrm d}\omega \;f_j(\omega ) |0\rangle \!\langle {\textrm{e}}_j| \otimes b^\dagger _{j}(\omega ) + \mathrm {h.c.}, \end{aligned}$$

with $|{\textrm{e}}_1\rangle ,\ldots ,|{\textrm{e}}_d\rangle \in \mathcal {H}_{{\textrm{e}}}$ being a collection of linearly independent vectors in ${\mathcal {H}}_{\textrm{e}}$, and where h.c. stands for the Hermitian conjugated term; each function $f_j(\omega )$ (form factor) modulates the coupling between the system and the *j*th mode of the bath. The boson creation and annihilation operators $b^\dagger _j(\omega )$ and $b_j(\omega )$ satisfy the standard canonical commutation relations: $[b_i(\omega ),b_{j}(\omega ')]=0$ and $[b_i(\omega ),b^\dag _{j}(\omega ')]=\delta _{ij}\delta (\omega -\omega ')$, to be interpreted in the distributional sense.

In the following, without loss of generality we will set the initial time of the evolution induced by ${\textbf {H}}$ as $t_0=0$ unless otherwise stated.

### Unitary and reduced dynamics

As discussed in^38^ (see also^35,46^ for the spin–boson case), the dynamics of an arbitrary state in the form

17 $$\begin{aligned} |\Psi _0\rangle = |\psi _0\rangle \otimes |{\textrm vac}\rangle ,\;\;\text {with}\quad |\psi _0\rangle =\alpha |0\rangle + |\psi _{{\textrm{e}}}\rangle ,\;\;\alpha \in {\mathbb {C}},\;|\psi _{\textrm{e}}\rangle \in \mathcal {H}_{{\textrm{e}}} \end{aligned}$$

induced by the Hamiltonian ${\textbf {H}}$ in Eqs. (15)–(16), can be computed exactly. One finds that the time evolved state $|\Psi _t\rangle :={\textrm{e}}^{-{\textrm i}t{\textbf{H}}}|\Psi _0\rangle$ has the following form:

18 $$\begin{aligned} |\Psi _t\rangle = \Big [\alpha |0\rangle + |\psi _{\textrm{e}}(t)\rangle \Big ] \otimes |{\textrm vac}\rangle + |0\rangle \otimes \sum _{j=1}^d \int {\textrm d}\omega \, \xi _j(t,\omega ) b^\dagger _j(\omega ) |{\textrm vac}\rangle , \end{aligned}$$

with $|\psi _{\textrm{e}}(t)\rangle \in \mathcal {H}_{{\textrm{e}}}$ and the wavefunctions $\xi _j(t,\omega )$ satisfying the following system of equations, equivalent to the Schrödinger equation generated by ${\textbf {H}}$:

19 $$\begin{aligned} {\textrm i}|{\dot{\psi }}_{\textrm{e}}(t)\rangle= & {} H_{\textrm{e}}|\psi _{\textrm{e}}(t)\rangle + \sum _{j=1}^d \int {\textrm d}\omega \, f^*_j(\omega ) \xi _j(t,\omega ) |{\textrm{e}}_j\rangle , \end{aligned}$$

20 $$\begin{aligned} {\textrm i}\, {\dot{\xi }}_j(t,\omega )= & {} \omega \,\xi _j(t,\omega ) + f_j(\omega ) \langle {\textrm{e}}_j|\psi _{\textrm{e}}(t)\rangle , \ \ \ j=1,\ldots ,d . \end{aligned}$$

Solving Eq. (20):

21 $$\begin{aligned} \xi _j(t,\omega ) = -{\textrm i}\int _0^t {\textrm d}s\,{\textrm{e}}^{-{\textrm i}\omega (t-s)} f_j(\omega ) \langle {\textrm{e}}_j|\psi _{\textrm{e}}(s)\rangle , \end{aligned}$$

and inserting the solution into Eq. (19), one obtains

22 $$\begin{aligned} |\psi _{\textrm{e}}(t)\rangle = {\textrm A}(t) |\psi _{\textrm{e}}\rangle , \end{aligned}$$

where the time-dependent operator ${\textrm A}(t)\in {\mathcal {B}}(\mathcal {H}_{{\textrm{e}}})$ satisfies the following non-local equation:

23 $$\begin{aligned} \text{i}{\dot{\text{A}}}(t) = H_\text{e}\text{A}(t) + \int_0^t \text{d}s\;\text{G}(t-s)\text{A}(s), \ \ \ \text{A}(0) = {\mathrm{I}}_\text{e}, \end{aligned}$$

and the corresponding memory kernel ${\textrm G}(t)\in {\mathcal {B}}(\mathcal {H}_{{\textrm{e}}})$ has the following form:

24 $$\begin{aligned} {\textrm G}(t) = -{\textrm i}\sum _{j=1}^d \int {\textrm d}\omega \,{\textrm{e}}^{-{\textrm i}\omega t} |f_j(\omega )|^2 |{\textrm{e}}_j\rangle \!\langle {\textrm{e}}_j| . \end{aligned}$$

By using these properties, one easily finds the corresponding reduced evolution of the state of the system:

25 $$\begin{aligned} |\psi _0\rangle \!\langle \psi _0| \rightarrow \Lambda _t(|\psi _0\rangle \!\langle \psi _0|) = \text{Tr}_{\rm B} \, |\Psi _t\rangle \!\langle \Psi _t|= & {} \text{A}(t) |\psi _\text{e}\rangle \!\langle \psi _e|\text{A}^\dagger (t) + \text{A}(t) |\psi _\text{e}\rangle \!\langle 0| + |0\rangle \!\langle \psi _\text{e}|\text{A}^\dagger (t) \nonumber \\ {}+ & {} |0\rangle \!\langle 0| \Big ( |\alpha |^2 + \langle \psi _\text{e}|\left({\mathrm{I}}_{\rm e} - \text{A}^\dagger (t)\text{A}(t)\right)|\psi _e\rangle \Big ). \end{aligned}$$

By construction, the map $\Lambda _t$ is completely positive and trace preserving (CPTP) for all $t \ge 0$, and satisfies $\Lambda _{t=0} = {\mathcal {I}}_S$.

From Eq. (25) we can readily reconstruct the evolution of a generic density operator $\rho$ by linearity. To this purpose, it will be convenient to adopt the following block representation:

26 $$\begin{aligned} \rho = \left( \begin{array}{cc} {\hat{\rho }}_{\textrm{e}}&{} |{\textbf{w}}\rangle \\ \langle {\textbf{w}}| &{} \rho _{\textrm g} \end{array} \right) , \end{aligned}$$

where ${\hat{\rho }}_{{\textrm{e}}}\in {\mathcal {B}}(\mathcal {H}_{{\textrm{e}}})$, $|{\textbf{w}}\rangle \in \mathcal {H}_{{\textrm{e}}}$, and $\rho _g\in {\mathbb {R}}$. With this representation, one finds the following expression for the reduced evolution:

27 $$\begin{aligned} \Lambda _t(\rho ) = \left( \begin{array}{cc} {\textrm A}(t){\hat{\rho }}_{\textrm{e}}{\textrm A}^\dagger (t) &{} {\textrm A}(t)|{\textbf{w}}\rangle \\ \langle {\textbf{w}}|{\textrm A}^\dagger (t) &{} \rho _{\textrm g}(t) \end{array} \right) , \end{aligned}$$

with $\rho _{\textrm g}(t) = {\textrm{Tr}}\,\rho - {\textrm{Tr}}( {\textrm A}(t) {\hat{\rho }}_{\textrm{e}}{\textrm A}^\dagger (t))$. It is therefore clear that the entire dynamical map $\Lambda _t$ is uniquely characterized by the operator ${\textrm A}(t)$, which we shall denote as the *survival amplitude operator*, since the quantity $\Vert {\textrm A}(t)|\psi _{\textrm{e}}\rangle \Vert ^2$ defines the probability that the initial state $|\psi _{\textrm{e}}\rangle \otimes |{\textrm vac}\rangle$ does not decay to the ground state at the time *t*. By construction, the following normalization condition holds:

28 $$\begin{aligned} {\textrm{Tr}}\left[ {\textrm A}(t)| \psi _{\textrm{e}} \rangle \!\langle \psi _{\textrm{e}} | {\textrm A}^\dag (t)\right] + \sum _{j=1}^d \Vert \xi _j(t)\Vert ^2 = {\textrm{Tr}}\,| \psi _{\textrm{e}} \rangle \!\langle \psi _{\textrm{e}} | , \end{aligned}$$

which accounts for the property ${\textrm{Tr}}\,\Lambda _t(\rho ) = {\textrm{Tr}}\,\rho$ ensuring the preservation of the trace.

The operator ${\textrm A}(t) : {\mathcal {H}}_{\textrm{e}}\rightarrow {\mathcal {H}}_{\textrm{e}}$ reduces to a single function *a*(*t*) in the qubit case and Eq. (27) reduces to the well-known amplitude-damping qubit channel,

29 $$\begin{aligned} \Lambda _t(\rho ) = \left( \begin{array}{cc} |a(t)|^2 {\rho }_{11} &{} a(t) \rho _{10} \\ a^*(t) \rho _{01} &{} \rho _{00} + (1-|a(t)|^2)\rho _{11} \end{array} \right) ; \end{aligned}$$

as such, we shall refer to $\Lambda _t$ as a *multilevel amplitude-damping channel*. We point out that other generalizations of the amplitude-damping channel were analyzed in Refs.^47–50^; besides, a family of quantum channels further generalizing the structure (27), labeled as *excitation-damping channels*, was recently introduced and studied in^51^.

The following simple characterization of positivity and complete positivity for channels in the form (27) was proven in^38^:

#### Proposition 3.1

(^38^) The dynamical map (27) is completely positive if and only if ${\textrm A}(t)$ is a contraction w.r.t. the operator norm, i.e. $\Vert {\textrm A}(t)\Vert _{{\textrm op}} \le 1$ for all $t \ge 0$. Besides, it is completely positive if and only if it is positive.

As such, channels as in Eq. (27) represent an interesting example of channels for which complete positivity and positivity coincide, another important instance being multilevel dephasing channels (or Hadamard channels)^52,55^.

### Markovianity and divisibility of the model

Recall that a dynamical map $\Lambda _t$ is divisible whenever for any $t \ge s$ one has $\Lambda _t = \Lambda _{t,s} \Lambda _s$ for some corresponding propagator $\Lambda _{t,s} : {\mathcal {B}}({\mathcal {H}}) \rightarrow {\mathcal {B}}({\mathcal {H}})$; it is CP-divisible when $\Lambda _{t,s}$ is CPTP and P-divisible when $\Lambda _{t,s}$ is positive and trace-preserving. CP-divisibility is a common core of the various (and generally inequivalent) definitions of *Markovianity* in open quantum systems. Clearly, every invertible map is also divisible with

30 $$\begin{aligned} \Lambda _{t,s} = \Lambda _t \Lambda _s^{-1}, \end{aligned}$$

and is thus CP-divisible (resp. P-divisible) if and only if the map in Eq. (30) is completely positive (resp. positive) for all $t\ge s$. In particular, invertible and differentiable channels satisfy the master equation ${{\dot{\Lambda }}}_t(\rho )={\mathcal {L}}_t\left( \Lambda _t(\rho )\right)$, with the time-local generator ${\mathcal {L}}_t={{\dot{\Lambda }}}_t\Lambda _t^{-1}$, and it is known that $\Lambda _t$ is CP-divisible if and only if ${\mathcal {L}}_t$ is a time-dependent Gorini–Kossakowski–Lindblad–Sudarshan (GKLS) map^53,54^.

In our case, a simple computation shows that the multilevel amplitude-damping channel $\Lambda _t$ as defined in Eq. (27) is invertible if and only if the corresponding survival amplitude operator ${\textrm A}(t)$ is invertible. In such a case, the channel is divisible with

31 $$\begin{aligned} \Lambda _{t,s}(\rho ) = \left( \begin{array}{cc} {\textrm A}(t,s){\hat{\rho }}_{\textrm{e}}{\textrm A}^\dagger (t,s) &{} {\textrm A}(t,s)|{\textbf{w}}\rangle \\ \langle {\textbf{w}}| {\textrm A}^\dagger (t,s) &{} \rho _{\textrm g}(t,s) \end{array} \right) \end{aligned}$$

where ${\textrm A}(t,s) = {\textrm A}(t){\textrm A}^{-1}(s)$ and $\rho _{\textrm g}(t,s) = {\textrm{Tr}}\rho - {\textrm{Tr}}[{\textrm A}(t,s){\hat{\rho }}_{\textrm{e}}{\textrm A}^\dagger (t,s)]$. This readily implies the following characterization of CP-divisibility and P-divisibility.

#### Proposition 3.2

(^38^) Let the amplitude operator ${\textrm A}(t)$ be invertible for all $t \ge 0$. Then $\Lambda _t$ is CP-divisible if and only if

32 $$\begin{aligned} \Vert {\textrm A}(t,s) \Vert _{{\textrm op}} \le 1 , \end{aligned}$$

for all $t \ge s$, and it is P-divisible if and only if it is CP-divisible.

Interestingly, CP-divisibility and P-divisibility are equivalent properties for the multilevel amplitude-damping channel. We also notice that the property (32) is equivalent, provided that $t\mapsto {\textrm A}(t)$ is differentiable, to the following one:

33 $$\begin{aligned} \frac{{\textrm d}}{{\textrm d}t}\,{\textrm{Tr}}\left[ {\textrm A}(t)| \psi _{\textrm{e}} \rangle \!\langle \psi _{\textrm{e}} | {\textrm A}^\dag (t)\right] \le 0 , \end{aligned}$$

for any $|\psi _{\textrm{e}}\rangle \in {\mathcal {H}}_{\textrm{e}}$. In other words:

#### Corollary 3.1

Let $\Lambda _t$ being invertible and differentiable. Then $\Lambda _t$ is a monotonic contraction w.r.t. trace norm in ${\mathcal {B}}({\mathcal {H}})$ if and only if ${\textrm A}(t)$ is a monotonic contraction in ${\mathcal {H}}_{\textrm{e}}$ (w.r.t. the natural norm induced by the inner product in ${\mathcal {H}}_{\textrm{e}}$).

The time-dependent generator associated with the channel reads

34 $$\begin{aligned} {\mathcal {L}}_t(\rho ) = \left( \begin{array}{cc} {\textrm L}(t){\hat{\rho }}_{\textrm{e}}+ {\hat{\rho }}_{\textrm{e}}{\textrm L}^\dagger (t) &{} {\textrm L}(t)|{\textbf{w}}\rangle \\ \langle {\textbf{w}}|{\textrm L}^\dagger (t) &{} -{\textrm{Tr}}({\hat{\rho }}_{\textrm{e}}[ {\textrm L}(t) + {\textrm L}^\dagger (t)]) \end{array} \right) , \end{aligned}$$

where

35 $$\begin{aligned} {\textrm L}(t) = {\dot{{\textrm A}}}(t){\textrm A}^{-1}(t) , \end{aligned}$$

i.e. ${\textrm A}(t)$ satisfies the following time-local dynamical equation

36 $$\begin{aligned} {\dot{\text{A}}}(t) = \text{L}(t)\text{A}(t) \ , \ \ \ \text{A}(0) ={\mathrm{I}}_\text{e}, \end{aligned}$$

and hence the corresponding propagator in ${\mathcal {H}}_{\textrm{e}}$ formally reads

37 $$\begin{aligned} {\textrm A}(t,s) = {\mathcal {T}}\exp \Bigl ( \int _{s}^t {\textrm L}(\tau )\,{\textrm d}\tau \Bigr ), \end{aligned}$$

with ${\mathcal {T}}$ signaling time ordering. Finally, by defining the time-dependent Hermitian operators

38 $$\begin{aligned} {\textrm H}_{\textrm{e}}(t) := \frac{{\textrm i}}{2} [{\textrm L}(t) - {\textrm L}^\dagger (t)],\qquad {\Gamma }(t) := - [{\textrm L}(t) + {\textrm L}^\dagger (t)] , \end{aligned}$$

the corresponding time-local generator can be written as

39 $$\begin{aligned} {\mathcal {L}}_t(\rho ) = -{\textrm i}[{\textrm H}_{\textrm{e}}(t),\rho ] - \frac{1}{2} \{ \Gamma (t), \rho \} + {\textrm{Tr}}(\Gamma (t) \rho ) \, |0\rangle \!\langle 0| , \end{aligned}$$

which is manifestly a GKLS generator provided that $\Gamma (t)\ge 0$, that is, the operator $\Gamma (t)$ is positive semidefinite at all times. Consequently,

#### Proposition 3.3

The reduced evolution induced by a multilevel spin–boson model is CP-divisible if and only if $\Gamma (t)\ge 0$ for all $t\ge 0$.

## The flat coupling limit

We shall focus hereafter on a particular choice of the form factors $f_j(\omega )$ modulating the coupling between the system and the *j*th mode of the field. Namely, we shall consider the case of *flat* couplings on all (positive and negative) values of $\omega$:

40 $$\begin{aligned} |f_j(\omega )|^2 = \frac{\gamma _j}{2\pi } , \quad -\infty \le \omega \le \infty , \end{aligned}$$

with $\gamma _j\ge 0$ being a coupling constant. This choice of coupling corresponds, in the position representation, to the case in which the coupling between the system and each mode of the field is described by a Dirac delta interaction; as such, it provides an idealized description of short-range system-field interactions. Clearly, such form factors are not normalizable, whence the self-adjointness of the corresponding multilevel spin–boson model is not guaranteed *a priori*; these mathematically issues are thoroughly discussed in greater generality in^44,46^. For our purposes, here it will suffice to observe that the equations of motion (19)–(20) induced by the model are indeed well-posed in this limit. Indeed, substituting Eq. (40) into the expression (24), the memory kernel ${\textrm G}(t)$ collapses into a Dirac delta:

41 $$\begin{aligned} {\textrm G}(t-s)=-{\textrm i}\delta (t-s)\sum _{j=1}^d\gamma _j| {\textrm{e}}_j \rangle \!\langle {\textrm{e}}_j | , \end{aligned}$$

that is, Eq. (23), and thus both Eqs. (19)–(20), are *memoryless*: they do not involve any dependence on past times.

### Dynamics and reduced dynamics

Specifically, Eq. (23) for the survival amplitude operator simplifies as follows:

42 $$\begin{aligned} {\textrm i}{{\dot{{\textrm A}}}}(t)=\left( {\textrm H}_e-\frac{{\textrm i}}{2}\Gamma \right) {\textrm A}(t), \end{aligned}$$

where

43 $$\begin{aligned} \Gamma = 2\sum _{j=1}^d \gamma _j | {\textrm{e}}_j \rangle \!\langle {\textrm{e}}_j | \ge 0 \end{aligned}$$

and its solution simply reads

44 $$\begin{aligned} {\textrm A}(t)={\textrm{e}}^{-t\left( {\textrm i}{\textrm H}_{\textrm{e}}+\frac{1}{2}\Gamma \right) }. \end{aligned}$$

Consequently, the solution of the system (19)–(20) reads as follows:

45 $$\begin{aligned} |\psi _{\textrm{e}}(t)\rangle= & {} {\textrm{e}}^{-t\left( {\textrm i}{\textrm H}_{\textrm{e}}+\frac{1}{2}\Gamma \right) }|\psi _{\textrm{e}}\rangle , \end{aligned}$$

46 $$\begin{aligned} {\xi }_j(t,\omega )= & {} -{\textrm i}\sqrt{\frac{\gamma _j}{2\pi }} \int _0^t {\textrm d}s \;{\textrm{e}}^{-{\textrm i}\omega (t-s)} \left\langle {\textrm{e}}_j\,\Bigg|\,{\textrm{e}}^{-s\left( {\textrm i}{\textrm H}_{\textrm{e}}+\frac{1}{2}\Gamma \right) }\Bigg|\,\psi _e\right\rangle . \end{aligned}$$

Two important remarks follow.

#### Wavefunction in the position representation

 Interestingly, Eq. (46) corresponds, in the position representation, to a compactly supported wavefunction. Indeed, defining the Fourier antitransform of $\xi _j(t,\omega )$ via ${\hat{\xi }}_j(t,x)=\int {\textrm d}\omega \;{\textrm{e}}^{{\textrm i}\omega x}\xi _j(t,\omega )$, one easily finds

47 $$\begin{aligned} {\hat{\xi }}_j(t,x) = -{\textrm i}\sqrt{2\pi \gamma _j} \int _0^t {\textrm d}s \left\langle {\textrm{e}}_j\,\Bigg|\,{\textrm{e}}^{-(t-x)\left( {\textrm i}{\textrm H}_{\textrm{e}}+\frac{1}{2}\Gamma \right) }\Bigg|\,\psi _e\right\rangle \, \chi _{[0,t]}(x) , \end{aligned}$$

where $\chi _I(x)$ is the characteristic function of the interval *I*. Hence, ${\hat{\xi }}_j(t,x)$ is entirely supported on [0, *t*], for positive times $t>0$. This result is analogous to the one obtained (in the single-atom case) in^36^ and has the following intuitive explanation: preparing the system in the state $|\psi \rangle \otimes |{\textrm vac}\rangle$ (multilevel atom in a generic state of the excited sector + all modes of the boson field in the vacuum state), the system will be in a superposition of a state in which no bosons are emitted, plus all possible states in which the system decays and a boson is emitted at the position $x=0$ in *only one* of all possible modes: for each mode, the emitted boson moves from left to right at unit speed (since we keep $c=1$), whence the wavefunction at time *t* will be supported at [0, *t*]. This property of $\xi _j(t,\omega )$ will be crucial to prove Prop. 4.1, which, in turn, will play important role in the analysis of Markovianity of the quantum measurement process in the next section.

#### Case

$[{\textrm H}_{\textrm{e}},\Gamma ]=0$. In this case the vectors $\{|{\textrm{e}}_j\rangle \}_j$ are *eigenvectors* of $\Gamma$ and thus ${\textrm H}_{\textrm{e}}=\sum _j\omega _j| {\textrm{e}}_j \rangle \!\langle e_j |$, whence Eqs. (45)–(46) read explicitly

48 $$\begin{aligned} |\psi _{\textrm{e}}(t)\rangle= & {} \sum _\ell \langle {\textrm{e}}_\ell |\psi _{\textrm{e}}\rangle {\textrm{e}}^{-t\left( {\textrm i}\omega _\ell +\frac{1}{2}\gamma _\ell \right) }|{\textrm{e}}_\ell \rangle , \end{aligned}$$

49 $$\begin{aligned} {\xi }_j(t,\omega )= & {} -{\textrm i}\sqrt{\frac{\gamma _j}{2\pi }} \langle {\textrm{e}}_j|\psi _{\textrm{e}}\rangle \int _0^t {\textrm d}s \;{\textrm{e}}^{-{\textrm i}\omega (t-s)} {\textrm{e}}^{-s\left( {\textrm i}\omega _j+\frac{1}{2}\gamma _j\right) }, \end{aligned}$$

that is, the survival operator is simply given by

50 $$\begin{aligned} {\textrm A}(t)=\sum _\ell {\textrm{e}}^{-t\left( {\textrm i}\omega _\ell +\frac{1}{2}\gamma _\ell \right) }| {\textrm{e}}_\ell \rangle \!\langle {\textrm{e}}_\ell | ; \end{aligned}$$

furthermore, in the position representation,

51 $$\begin{aligned} {\hat{\xi }}_j(t,x) = -{\textrm i}\sqrt{2\pi \gamma _j}\langle {\textrm{e}}_j|\psi _{\textrm{e}}\rangle \int _0^t {\textrm d}s\;{\textrm{e}}^{-(t-x)\left( {\textrm i}\omega _j+\frac{1}{2}\gamma _j\right) }\, \chi _{[0,t]}(x). \end{aligned}$$

In particular, if $|\psi _{\textrm{e}}\rangle =|{\textrm{e}}_\alpha \rangle$ for some fixed $\alpha =1,\ldots ,d$, then

52 $$\begin{aligned} |\psi _{\textrm{e}}(t)\rangle= & {} {\textrm{e}}^{-t\left( {\textrm i}\omega _\alpha +\frac{1}{2}\gamma _\alpha \right) }|{\textrm{e}}_\alpha \rangle , \end{aligned}$$

53 $$\begin{aligned} {\xi }_j(t,\omega )= & {} -{\textrm i}\sqrt{\frac{\gamma _j}{2\pi }} \delta _{j\alpha }\int _0^t {\textrm d}s\;{\textrm{e}}^{-(t-x)\left( {\textrm i}\omega _j+\frac{1}{2}\gamma _j\right) }\, \chi _{[0,t]}(x), \end{aligned}$$

that is, the evolution of the system only involves $|{\textrm{e}}_\alpha \rangle$ and the $\alpha$th mode of the boson field, effectively behaving as a spin–boson model. The multilevel, multimode nature of the model only emerges when taking into account different initial states.

As a final observation, notice that the equality $[{\textrm H}_{\textrm{e}},\Gamma ]=0$ holds if and only if ${\textrm A}(t)$ is a *normal* matrix, i.e. $[{\textrm A}(t),{\textrm A}^\dag (t)]=0$. Indeed, by the properties of matrix exponentials,

54 $$\begin{aligned}{}[{\textrm A}(t),{\textrm A}^\dag (t)]=0\;\;\forall t\in {\mathbb {R}}\iff 0=\left[ {\textrm i}{\textrm H}_{\textrm{e}}+\frac{1}{2}\Gamma ,-{\textrm i}{\textrm H}_{\textrm{e}}+\frac{1}{2}\Gamma \right] ={\textrm i}[{\textrm H}_{\textrm{e}},\Gamma ]. \end{aligned}$$

Finally, as for the reduced dynamics, the corresponding channel $\Lambda _t$ is a *semigroup*, i.e. $\Lambda _{t}=\Lambda _{t-s}\Lambda _s$, as can be either seen by directly substituting Eq. (44) into the expression (27) of the channel or, equivalently, by simply observing that the expression (34) of its time-local generator reduces to

55 $$\begin{aligned} {\mathcal {L}}_t(\rho )\equiv {\mathcal {L}}(\rho )= & {} -{\textrm i}[{\textrm H}_{\textrm{e}},\rho ] - \frac{1}{2} \{ \Gamma , \rho \}+ {\textrm{Tr}}(\Gamma \rho ) \, |0\rangle \!\langle 0| \nonumber \\= & {} -{\textrm i}[{\textrm H}_{\textrm{e}},\rho ] + \sum _{j=1}^d \gamma _j \Big ( |0\rangle \!\langle {\textrm{e}}_j|\rho |{\textrm{e}}_j\rangle \!\langle 0| - \frac{1}{2} \{ |{\textrm{e}}_j\rangle \!\langle {\textrm{e}}_j|,\rho \} \Big ), \end{aligned}$$

which is clearly a time-independent GKLS generator. *A fortiori*, the channel is CP-divisible at all times.

### Other properties

In general, as discussed, the dynamics induced by ${\textbf {H}}$ on states with a number of excitations *larger* than one is not solvable. For instance, the evolution of states with two excitations, like

56 $$\begin{aligned} {\textrm{e}}^{-{\textrm i}t{\textbf {H}}}|\psi _{\textrm{e}}\rangle \otimes b_j^\dag (\eta )|{\textrm vac}\rangle \quad \text {or}\quad {\textrm{e}}^{-{\textrm i}t{\textbf {H}}}|0\rangle \otimes b_j^\dag (\eta )b_{j'}^\dag (\eta ')|{\textrm vac}\rangle , \end{aligned}$$

where we used the compact notation

57 $$\begin{aligned} b^\dag _j\left( \eta \right) =\int {\textrm d}\omega \;\eta (\omega )b_j^\dag (\omega ), \end{aligned}$$

cannot be computed explicitly. This, in particular, would generally prevent us from computing the joint probability distributions associated with the quantum process obtained by repeatedly probing the system via any orthonormal basis: a measurement in a basis which is not compatible with the $\mathcal {H}_{{\textrm{e}}}\oplus \mathcal {H}_{\textrm g}$ splitting, as we will see, will necessarily involve taking into account higher-excitation sectors.

However, *specifically* in the flat coupling case, a fundamental property holds:

#### Proposition 4.1

For $\xi _j(t,\omega )$ defined in Eq. (46), one has

58 $$\begin{aligned} U_{\tau } \,{\mathrm{I}}_{\rm S} \otimes b^\dagger _j(\xi _j(t)) \,U^\dagger _{\tau } = {\mathrm{I}}_{\rm S} \otimes b^\dagger _j(\xi _j(\tau ,t)) , \end{aligned}$$

for any $\tau \ge 0$, where we define

59 $$\begin{aligned} \xi _j(\tau ,t,\omega ) :={\textrm{e}}^{-{\textrm i}\omega \tau }\xi _j(t,\omega ). \end{aligned}$$

The proof of Prop. 4.1 is reported in the Supplementary Information. This property will be used in the following section to compute the multitime statistics associated with the process both with respect to measurements in bases compatible with the $\mathcal {H}_{{\textrm{e}}}\oplus \mathcal {H}_{\textrm g}$ splitting (cf. Sect. 5.1) and arbitrary bases (cf. Sect. 5.2).

## Markovianity and classicality

The limiting choice of flat couplings in the interaction Hamiltonian (16) is the only possible choice ensuring that the reduced dynamics, associated with the map $\Lambda _t$, is a GKLS semigroup—historically, such semigroups have long been denoted as *Markovian semigroups*. However, the semigroup property by itself does *not* suffices, in general, to ensure Markovianity of the corresponding process, in the sense of Eq. (3), with respect to an arbitrary sharp measurement.

In this regard, we shall consider projective measurements w.r.t. an orthonormal basis $\{|x_\alpha \rangle \}_{x=0,\ldots ,d}$ in the Hilbert space ${\mathcal {H}}_{{\textrm{S}}}$. We shall assume the initial state of the system+bath to be $|\psi _0\rangle \otimes |{\textrm vac}\rangle$. With this choice, the multitime statistic generated by this process, cf. Eq. (7), is

60 $$\begin{aligned} {\mathbb {P}}_n(x_n,t_n;\ldots ;x_1,t_1) = {\textrm{Tr}} [ {\mathcal {P}}_{x_n}\otimes {\mathcal {I}}_{\rm B} \circ U_{t_n-t_{n-1}} \ldots \circ {\mathcal {U}}_{t_2-t_1} \circ {\mathcal {P}}_{x_1}\otimes {\mathcal {I}}_{\rm B} \circ U_{t_1}(|\psi _0\rangle \!\langle \psi _0| \otimes |{\textrm vac}\rangle \!\langle {\textrm vac}|)] , \end{aligned}$$

where $${\mathcal {P}}_x(X) = P_x X P_x$$, $$P_x=| x \rangle \!\langle x |$$.

In this section we will provide an explicit proof of the fact that, indeed, this quantum process is Markovian in the limit of flat coupling. Furthermore, necessary and sufficient conditions for the classicality of the model—that is, for the Chapman–Kolmogorov Eq. (6) to be satisfied—will be found. For the sake of simplicity, we shall start by examining the situation in which the measurements are performed with respect to an orthonormal basis compatible with the splitting $\mathcal {H}_{{\textrm{S}}}=\mathcal {H}_{\textrm{e}}\oplus \mathcal {H}_{\textrm g}$, and then examine the general situation.

### Measurements in bases compatible with the ${\mathcal {H}}_{\textrm g}\oplus {\mathcal {H}}_{\textrm{e}}$ splitting

Consider a projective measurements w.r.t. an orthonormal basis *compatible* with the splitting of the Hilbert space $\mathcal {H}_{{\textrm{S}}}$ of the system into its excited and ground sector, that is, a basis $\{|x\rangle \}_{x=0,\ldots ,d}$ such that $|0\rangle$ is the ground state of the atom and $\{|x\rangle \}_{x=1,\ldots ,d}\subset \mathcal {H}_{{\textrm{e}}}$ is any orthonormal basis of the excited sector. Let us compute the corresponding *n*-point joint probability. Assuming the following initial state $|\Psi _0\rangle = |\psi \rangle \otimes |{\textrm vac}\rangle$, with $|\psi \rangle = \alpha |0\rangle \oplus |\psi _{\textrm{e}}\rangle$, and using hereafter the compact notation (57) for an arbitrary wavefunction $\eta$, one finds, as discussed in Sect. 4,

61 $$\begin{aligned} U_{t_1}|\Psi _0\rangle = \Big [ \alpha |0\rangle \otimes |{\textrm vac}\rangle + {\textrm A}(t_1)|\psi _{\textrm{e}}\rangle \Big ] \otimes |{\textrm vac}\rangle + |0\rangle \otimes \sum _{j=1}^d b_j^\dagger (\xi _j(t_1))|{\textrm vac}\rangle , \end{aligned}$$

with ${\textrm A}(t)$ as in Eq. (44) and $\xi _j(t)$ as in Eq. (46); hence,

62 $$\begin{aligned} P_{x_1} \otimes \mathrm{I}_{\rm B}\, U_{t_1}|\Psi _0\rangle= & {} \langle x_1|{\textrm A}(t_1)|\psi _{\textrm{e}}\rangle \, |x_1\rangle \otimes |{\textrm vac}\rangle , \end{aligned}$$

63 $$\begin{aligned} P_0 \otimes \mathrm{I}_{\rm B}\, U_{t_1}|\Psi _0\rangle= & {} |0\rangle \otimes \Big (\alpha |{\textrm vac}\rangle + \sum _{j=1}^{d} b_j^\dagger (\xi _j(t_1))|{\textrm vac}\rangle \Big ) . \end{aligned}$$

Therefore, we have

64 $$\begin{aligned} {\mathbb {P}}_1(x_1,t) = | \langle x_1|{\textrm A}(t)|\psi _{\textrm{e}}\rangle |^2 \ , \ \ \ {\mathbb {P}}_1(0,t) = |\alpha |^2 + \sum _{j=1}^{d} \Vert \xi _j(t)\Vert ^2 . \end{aligned}$$

Now we must apply the second unitary evolution $U_{\Delta t_2}$. We have

65 $$\begin{aligned} U_{\Delta t_2} P_{x_1} \otimes \mathrm{I}_{\rm B}\, U_{t_1}|\Psi _0\rangle= & {} \langle x_1|{\textrm A}(t_1)|x_1\rangle \,U_{\Delta t_2}\left( |x_1\rangle \otimes |{\textrm vac}\rangle \right) \nonumber \\= & {} \langle x_1|{\textrm A}(t)|\psi _{\textrm{e}}\rangle \Big ( {\textrm A}(\Delta t_2) |x_1\rangle \otimes |{\textrm vac}\rangle + |0\rangle \otimes \sum _{j=1}^d b_j^\dagger (\eta ^{(1)}_j(\Delta t_2))|{\textrm vac}\rangle \Big ) , \end{aligned}$$

where $\Delta t_k = t_k - t_{k-1}$, with the function $\eta ^{(\alpha )}_j(\tau )$ being defined as follows:

66 $$\begin{aligned} \eta ^{(\alpha )}_j(\tau ,\omega ) = -i\int _0^\tau {\textrm d}s\;{\textrm{e}}^{-{\textrm i}\omega (\tau -s)} f_j(\omega ) \langle {\textrm{e}}_j|{\textrm A}(s)|x_\alpha \rangle , \end{aligned}$$

and we used again the explicit form of the evolution in the single-excitation sector. Besides, using Prop. 4.1,

67 $$\begin{aligned} U_{\Delta t_2} P_0 \otimes \mathrm{I}_{\rm B}\, U_{t_1}|\Psi _0\rangle= & {} U_{\Delta t_2}\left[ |0\rangle \otimes \Big (\alpha |{\textrm vac}\rangle + \sum _{j=1}^{d} b_j^\dagger (\xi _j(t_1))|{\textrm vac}\rangle \Big ) \right] \nonumber \\ {}= & {} |0\rangle \otimes \Big (\alpha |{\textrm vac}\rangle + \sum _{j=1}^{d} b_j^\dagger (\xi _j(\Delta t_2,t_1))|{\textrm vac}\rangle \Big ) , \end{aligned}$$

where $\xi _j(\tau ,t,\omega ) = {\textrm{e}}^{-{\textrm i}\omega \tau } \xi _j(t,\omega )$. Notice that, differently from all previous steps, this is the *only* step that holds specifically for the flat coupling case.

The second projective measurement gives rise to

68 $$\begin{aligned} P_{x_2} \otimes \mathrm{I}_{\rm B}\, U_{\Delta t_2} P_{x_1} \otimes \mathrm{I}_{\rm B}\, U_{t_1}|\Psi _0\rangle= & {} \langle x_2|{\textrm A}(\Delta t_2)|x_1\rangle \!\langle x_1|{\textrm A}(t_1)|\psi _{\textrm{e}}\rangle \, |x_2\rangle \otimes |{\textrm vac}\rangle , \end{aligned}$$

69 $$\begin{aligned} P_{0} \otimes \mathrm{I}_{\rm B}\, U_{\Delta t_2} P_{x_1} \otimes \mathrm{I}_{\rm B}\, U_{t_1}|\Psi _0\rangle= & {} \langle x_1|{\textrm A}(t_1)|\psi _{\textrm{e}}\rangle \, |0\rangle \otimes \sum _{j=1}^d b_j^\dagger (\eta ^{(1)}(\Delta t_2))|{\textrm vac}\rangle , \end{aligned}$$

70 $$\begin{aligned} P_{x_2} \otimes \mathrm{I}_{\rm B}\, U_{\Delta t_2} P_{0} \otimes \mathrm{I}_{\rm B}\, U_{t_1}|\Psi _0\rangle= & {} 0 , \end{aligned}$$

71 $$\begin{aligned} P_{0} \otimes \mathrm{I}_{\rm B}\, U_{\Delta t_2} P_{0} \otimes \mathrm{I}_{\rm B}\, U_{t_1}|\Psi _0\rangle= & {} |0\rangle \otimes \Big (\alpha |{\textrm vac}\rangle + \sum _{k=1}^{d} b_j^\dagger (\xi _j(\Delta t_2,t_1))|{\textrm vac}\rangle \Big ) . \end{aligned}$$

One finds

72 $$\begin{aligned} {\mathbb {P}}_2(x_2,t_2;x_1,t_1)= & {} |\langle x_2|{\textrm A}(\Delta t_2)|x_1\rangle |^2 \, {\mathbb {P}}_1(x_1,t_1) \end{aligned}$$

73 $$\begin{aligned} {\mathbb {P}}_2(0,t_2;x_1,t_1)= & {} \Vert \eta ^{(1)}_{x_1}(\Delta t_2)\Vert ^2 \, {\mathbb {P}}_1(x_1,t_1) \end{aligned}$$

74 $$\begin{aligned} {\mathbb {P}}_2(x_2,t_2;0,t_1)= & {} 0 , \end{aligned}$$

75 $$\begin{aligned} {\mathbb {P}}_2(0,t_2;0,t_1)= & {} {\mathbb {P}}_1(0,t_1) . \end{aligned}$$

It is straightforward to generalize the above procedure for *n*-point joint probabilities:

76 $$\begin{aligned} {\mathbb {P}}_n(x_n,t_n;x_{n-1},t_{n-1};\ldots ;x_1,t_1)= & {} |\langle x_{n}|{\textrm A}(\Delta t_n)|x_{n-1}\rangle |^2 \ldots |\langle x_2|{\textrm A}(\Delta t_2)|x_1\rangle |^2 \, {\mathbb {P}}_1(x_1,t_1) , \end{aligned}$$

77 $$\begin{aligned} {\mathbb {P}}_n(0,t_n;\ldots ;0,t_{k+1};x_{k},t_{k};\ldots ;x_1,t_1)= & {} \Vert \eta ^{(k)}_{x_k}(\Delta t_{k+1})\Vert ^2 \, {\mathbb {P}}_k(x_k,t_k;x_{k-1},t_{k-1};\ldots ;x_1,t_1) , \end{aligned}$$

78 $$\begin{aligned} {\mathbb {P}}_n(0,t_n;0,t_{n-1};\ldots ;0,t_1)= & {} {\mathbb {P}}_1(0,t_1) , \end{aligned}$$

and all remaining probabilities vanish. Importantly, all joint probabilities associated to phenomena in which the system is *first* measured in its ground state ($x_j=0$) and *later* in an excited state ($x_{j+1}\ne 0$) vanish: physically, because of our particular choice of form factors, emitted photons cannot be reabsorbed—the decay of the system is irreversible.

#### Markovianity

Having computed the full family of joint probabilities associated with the system, we can now investigate its Markovianity. A direct scrutiny of Eqs. (76)–(78) shows that the process is indeed Markovian:

##### Proposition 5.1

Let ${\textbf {H}}$ be a multilevel spin–boson model in the limit of flat coupling, $|\psi _0\rangle =|\psi _{\textrm{e}}\rangle +\alpha |0\rangle \in \mathcal {H}_{{\textrm{S}}}$, and $\{|x\rangle \}_{x=1,\ldots ,d}\subset \mathcal {H}_{{\textrm{S}}}$ any orthonormal basis of the excited sector $\mathcal {H}_{{\textrm{e}}}$. Then the process defined by Eq. (60) is a Markovian process with 1-time probability ${\mathbb {P}}_1(x,t)$ and transition probabilities ${\mathbb {P}}(x,t|y,s)$ given as follows: for any $x=1,\ldots ,d$ and $t\ge s\ge 0$,

79 $$\begin{aligned} {\mathbb {P}}_1(x,t)= & {} \left| \langle x|{\textrm A}(t)|\psi _{\textrm{e}}\rangle \right| ^2, \end{aligned}$$

80 $$\begin{aligned} {\mathbb {P}}_1(0,t)= & {} 1-\sum _{x=1}^d\left| \langle x|{\textrm A}(t)|\psi _{\textrm{e}}\rangle \right| ^2 \end{aligned}$$

and

81 $$\begin{aligned} {\mathbb {P}}(x,t|y,s)= & {} | \langle x|{\textrm A}(t-s)|y\rangle |^2 , \end{aligned}$$

82 $$\begin{aligned} {\mathbb {P}}(0,t|y,s)= & {} 1-\sum _{x=1}^d | \langle x|{\textrm A}(t-s)|y\rangle |^2, \end{aligned}$$

83 $$\begin{aligned} {\mathbb {P}}(x,t|0,s)= & {} 0 , \end{aligned}$$

84 $$\begin{aligned} {\mathbb {P}}(0,t|0,s)= & {} 1 . \end{aligned}$$

Finally, the process satisfies the regression equality (10).

##### Proof

Eqs. (79)–(80) simply coincide with Eq. (64) taking into account the unitarity of the evolution induced by ${\textbf {H}}$; similarly, Eqs. (81)–(84) follow from Eq. (76)–(78) and, again, the unitarity of the evolution. Moreover, recalling the definition of the reduced dynamics $\Lambda _t$ and the corresponding propagator, we indeed have, for all $x,y=0,1,\ldots ,d$,

85 $$\begin{aligned} {\mathbb {P}}(x,t|y,s) =\langle x| \Lambda _{t,s}(|y\rangle \!\langle y|)| x\rangle , \end{aligned}$$

whence the final claim immediately follows from Prop. 2.1. $\square$

It is important to notice that these properties hold regardless of whether the orthonormal basis $\{|x\rangle \}_{x=1,\ldots ,d}\subset \mathcal {H}_{{\textrm{e}}}$ of the excited sector coincides with the “natural” basis $\{|{\textrm{e}}_x\rangle \}_{x=1,\ldots ,d}$ in which the operator $\Gamma$ is diagonal—what is really important is the fact that, choosing an orthonormal basis of the excited sector as our measurement basis, we are forced to ”complete” it with the ground state $|0\rangle$ of the system, thus obtaining an orthonormal basis of $\mathcal {H}_{{\textrm{S}}}$ which is compatible with the excited-ground splitting. The interpretation of Eqs. (81)–(84) is also immediate: if the system is found in its ground state $|0\rangle$ after some measurement, then it will be found in such a state in all future measurements. This is ultimately a consequence of the fact that, with our choice of measurement basis, the global state of the system+bath is never going to leave the excited sector. Also notice that, by Eq. (81), the transition probability ${\mathbb {P}}(x,t|y,s)$ for $x,y=1,\ldots ,d$ (that is, the probability associated with a transition $|y\rangle \rightarrow |x\rangle$ between two states of the excited sector) simply coincides with the squared modulus of the matrix element of ${\textrm A}(t-s)$ between such states.

#### Classicality

Let us now examine whether the process is classical, i.e., whether it satisfies the consistency conditions (2); since the process is Markovian, this happens if and only if the Chapman–Kolmogorov Eqs. (5)–(6) for the transition probability is satisfies by the conditional probabilities (81)–(84). For the multilevel spin–boson model it means that for all $x,y=1,\ldots ,d$ and $t\ge r\ge s\ge 0$,

86 $$\begin{aligned} {\mathbb {P}}(x,t|y,s)= & {} \sum _{z=0}^d{\mathbb {P}}(x,t|z,r){\mathbb {P}}(z,r|y,s); \end{aligned}$$

87 $$\begin{aligned} {\mathbb {P}}(0,t|y,s)= & {} \sum _{z=0}^d{\mathbb {P}}(0,t|z,r){\mathbb {P}}(z,r|y,s); \end{aligned}$$

88 $$\begin{aligned} {\mathbb {P}}(x,t|0,s)= & {} \sum _{z=0}^d{\mathbb {P}}(x,t|z,r){\mathbb {P}}(z,r|0,s); \end{aligned}$$

89 $$\begin{aligned} {\mathbb {P}}(0,t|0,s)= & {} \sum _{z=0}^d{\mathbb {P}}(0,t|z,r){\mathbb {P}}(z,r|0,s). \end{aligned}$$

By Eqs. (81)–(84), (88)–(89) reduce to the identities $0=0$ and $1=1$, but Eqs. (86)–(87) are not trivial and are not generally satisfied.

As it turns out, classicality *does* depend on the choice of measurement basis.

##### Proposition 5.2

Consider the same process as before with the additional condition $[{\textrm H}_{\textrm{e}},\Gamma ]=0$. Then:

- if $|x\rangle =|{\textrm{e}}_x\rangle$ for all $x=1,\ldots ,d$, then the process is classical;
- suppose that the spectrum of $\Gamma$ is nondegenerate, i.e. $\gamma _j\ne \gamma _\ell$ for $j\ne \ell$. Then the process is classical *if and only if* the chosen measurement basis is such that, for all $x=1,\ldots ,d$, $|x\rangle =|{\textrm{e}}_x\rangle$ possibly up to a phase shift.

The proof of this statement is reported in the Supplementary Information; we shall discuss here the meaning of this statement. When $[H_{\textrm{e}},\Gamma ]=0$, the basis $\{|{\textrm{e}}_j\rangle \}_{j=1,\ldots ,d}$ of eigenvectors of the decay operator $\Gamma$ is also an eigenbasis for $H_{\textrm{e}}$. Consequently, as discussed in the previous section, the evolution of the system does not “mix” the various eigenstates nor the modes of the boson field, whence the system effectively behaves as a two-level system interacting with a single bosonic mode. In this situation, a simple computation shows that both Chapman–Kolmogorov equations are satisfied. The less trivial part of the proof of the statement above is to show that, indeed, this is the *only* scenario in which classicality holds: any other measurement basis will cause nonclassical effects to emerge.

It is instructive to investigate this phenomenon at the light of the results of^3,4^ about the classicality of quantum Markov processes, which we briefly summarized in the Introduction. To this purpose, let us recall their results in a more precise way. A family of propagators is said to be non-coherence-generating-and-detecting (NCGD) with respect to a basis $\{|x\rangle \}_{x=0,\ldots ,d}$ of the system Hilbert space if it satisfies the following equality:

90 $$\begin{aligned} \Delta \circ \Lambda _{t_{k+1},t_k} \circ \Delta \circ \Lambda _{t_{k},t_{k-1}} \circ \Delta = \Delta \circ \Lambda _{t_{k+1},t_{k-1}} \circ \Delta , \end{aligned}$$

where

91 $$\begin{aligned} \Delta = \sum _{x=0}^d {\mathcal {P}}_x ,\qquad {\mathcal {P}}_x=| x \rangle \!\langle x | \cdot | x \rangle \!\langle x | , \end{aligned}$$

is a quantum channel representing perfect decoherence w.r.t. $\{|0\rangle ,|x\rangle _{x=1}^d\}$. Eq. (90) essentially means that, at any intermediate time $t_k$ between any couple of measurements at two times $t_{k+1}$ and $t_{k-1}$, not doing any measure—that is, applying the identity map—is indistinguishable from performing a measurement and then averaging on all results—that is, applying $\Delta$. In its essence, this condition may be regarded as the transposition of the requirement (2) from the level of multitime statistics to the underlying quantum channels which realize the statistics; this is essentially the content of the following characterization:

##### Theorem 5.1

(^4^, Theorem 1) Consider a family of joint probabilities $\{{\mathbb {P}}_n\}_{n}$ satisfying the Markov property. Then the family is classical if and only if there exist, on a suitable Hilbert space $\mathcal {H}$,

- a basis $$\{|x\rangle \}_{x=0,\ldots ,d}$$;
- a state ${\tilde{\rho }}_{0}\in {\mathcal {B}}(\mathcal {H})$ which is diagonal in said basis;
- a family of quantum channels ${\tilde{\Lambda }}_{t_{j+1},t_j}$ which are NCGD with respect to said basis,

such that

92 $$\begin{aligned} {\mathbb {P}}_n(x_n,t_n;\dots ;x_1,t_1)={\textrm{Tr}}\left[ {\mathcal {P}}_{x_n}{\tilde{\Lambda }}_{t_n,t_{n-1}}\cdots {\mathcal {P}}_{x_1}{\tilde{\Lambda }}_{t_1}({\tilde{\rho }}_{0})\right] . \end{aligned}$$

*Apparently*, the theorem above may seem in contrast with the content of Prop. 5.2, since the initial state assumed in our model, $\rho _{0}=| \psi _{\textrm{e}} \rangle \!\langle \psi _{\textrm{e}} |$, is generally not diagonal in the measurement basis. In fact, this is not the case. As thoroughly discussed in^4^, the maps ${\tilde{\Lambda }}_{t,s}$ and the initial state $\rho _{0}$ define an *artificial* dynamics of the system which, for a multitime statistics obtained by a preexisting underlying quantum system, does *not* generally coincide with the actual reduced dynamics nor the actual initial state, while reproducing the same statistics.

This can be better understood by looking at the explicit construction of ${\tilde{\Lambda }}_{t_{j+1},t_j}$ and ${\tilde{\rho }}_{0}$ for a given multitime statistics provided in^4^. Setting $t_1=0$, they define the channels ${\tilde{\Lambda }}_{t_{j+1},t_j}$ and the initial state ${\tilde{\rho }}_{0}$ via

93 $$\begin{aligned} {\tilde{\Lambda }}_{t_{j+1},t_j}\left( | x_j \rangle \!\langle y_j | \right)= & {} \delta _{x_j,y_j}\sum _{x_{j+1}=0}^d{\mathbb {P}}\left( x_{j+1},t_{j+1}|x_j,t_j\right) | x_j \rangle \!\langle x_j | ; \end{aligned}$$

94 $$\begin{aligned} {\tilde{\rho }}_{0}= & {} \sum _{x_1=0}^d{\mathbb {P}}_1(x_1,t_1)| x_1 \rangle \!\langle x_1 | , \end{aligned}$$

and clearly, in our case, neither ${\tilde{\Lambda }}_{t_{j+1},t_j}$ nor ${\tilde{\rho }}_{0}$ coincide with the actual reduced dynamics of the model nor the initial state. However, it is worth noticing that the actual propagators $\Lambda _{t_j,t_{j+1}}$ are indeed NCDG in our case.

Summing up: the multilevel spin–boson model ${\textbf {H}}$, repeatedly probed via sharp measurements associated via an orthonormal basis compatible with the $\mathcal {H}_{{\textrm{e}}}\oplus \mathcal {H}_{\textrm g}$ splitting, is Markovian—in the sense adopted in this paper—in the limit of flat couplings. Besides, while generally non-classical, the process becomes classical if $[{\textrm H}_{\textrm{e}},\Gamma ]=0$ *and* the chosen measurement basis coincides with the common basis of eigenvectors of ${\textrm H}_{\textrm{e}}$ and $\Gamma$. Roughly speaking, this happens because, when adopting this particular basis, no probability exchange between states competing with different eigenvectors $|{\textrm{e}}_j\rangle$ happens—no fundamentally quantum feature is unveiled in the measurement. Choosing any other basis will reveal the nonclassicality of the process.

### Measurements in arbitrary bases

Consider now a projective measurements w.r.t. to an arbitrary orthonormal basis $\{|x_\alpha \rangle \}_{\alpha =0,\ldots ,d}$ in ${\mathcal {H}}$. With this choice, it is no longer true that the entire process is constrained in the single-excitation sector: in fact, after the *n*th measurement, the following vector

95 $$\begin{aligned} |\Psi ^{x_n,\ldots ,x_1}_{t_n,\ldots ,t_1}\rangle = P_{x_n} \otimes \mathrm{I}_{\rm B}\, U_{\Delta t_{n}} \ldots U_{\Delta t_2} P_{x_1} \otimes \mathrm{I}_{\rm B}\, U_{t_1} |\Psi _0\rangle , \end{aligned}$$

belongs to the $(n+1)$-excitation sector. This fact makes the analysis quite technical and we present all the necessary details in the Supplementary Information. Evidently, the process is not classical; however, it is still Markovian.

#### Theorem 5.2

The process defined by

96 $$\begin{aligned} {\mathbb {P}}_n(x_n,t_n;\ldots ;x_1,t_1) = \Vert \, |\Psi ^{x_n,\ldots ,x_1}_{t_n,\ldots ,t_1}\rangle \, \Vert ^2 \end{aligned}$$

satisfies the regression equality (10) for an arbitrary orthonormal basis $\{|x_\alpha \rangle \}_{\alpha =0,\ldots ,d}\subset \mathcal {H}_{\textrm{e}}\oplus \mathcal {H}_{\textrm g}$.

The proof, reported in the Supplementary Information, is based on the following observation: any basis vector $|x_\alpha \rangle$ may be uniquely decomposed as $|x_\alpha \rangle = \mu _\alpha |0\rangle \oplus |{\tilde{x}}_\alpha \rangle$ with $|{\tilde{x}}_\alpha \rangle \in {\mathcal {H}}_{\textrm{e}}$. One has

97 $$\begin{aligned} U_{t}|{\tilde{x}}_\alpha \rangle \otimes |{\textrm vac}\rangle = {\textrm A}(t)|{\tilde{x}}_\alpha \rangle \otimes |{\textrm vac}\rangle + |0\rangle \otimes \sum _{j=1}^d b_j^\dagger ({\tilde{\eta }}^{(\alpha )}_j(t))|{\textrm vac}\rangle , \end{aligned}$$

where the function $\eta ^{(\alpha )}_j(\tau )$ is defined via

98 $$\begin{aligned} {\tilde{\eta }}^{(\alpha )}_j(t,\omega ) = -i\int _0^\tau {\textrm d}\omega \;{\textrm{e}}^{-{\textrm i}\omega (t-s)} f_j(\omega ) \langle {\textrm{e}}_j|{\textrm A}(s)|{\tilde{x}}_\alpha \rangle , \ \ \ j=1,\ldots ,d . \end{aligned}$$

Similarly, let us define

99 $$\begin{aligned} {\tilde{\eta }}^{(\alpha )}_j(\tau ,t,\omega ) = {\textrm{e}}^{-{\textrm i}\omega \tau } {\tilde{\eta }}^{(\alpha )}_j(t,\omega ) . \end{aligned}$$

With these definitions, the proof is based on two technical properties that hold specifically in the flat coupling limit, the first being Prop. 4.1, and the second one, again shown in the Supplementary Information, being the following one:

#### Proposition 5.3

The following property holds:

100 $$\begin{aligned}{} & {} \left\| \, b^\dagger _{j_1}(\xi _{j_1}(t_{n+1}-t_1,\Delta t_1)) \, \prod _{k=2}^n \sum _{j_k=1}^d b^\dagger _{j_k}({\tilde{\eta }}_{j_k}^{(k-1)}(t_{n+1}-t_k,\Delta t_k)) |{\textrm vac}\rangle \right\| ^2 \nonumber \\{} & {} = \left\| b^\dagger _{j_1}(\xi _{j_1}(\Delta t_1))\right\| ^2 \, \prod _{k=2}^n \left\| \sum _{j_k=1}^d b^\dagger _{j_k}({\tilde{\eta }}_{j_k}^{(k-1)}(t_{n+1}-t_k,\Delta t_k)) \right\| ^2 , \end{aligned}$$

for any $$n\ge 1$$.

The above property allows to prove the regression equality (10) (cf. Supplementary Information) and thus Markovianity.

#### Remark 5.1

Actually, the proof of Theorem 5.2 shows that the regression equality (10) holds for a much more general measurement strategy, that is, without using the same fixed orthonormal basis to measure the system at $\{t_1,t_2,\ldots \}$, but rather using at each moment $t_k$ any arbitrary orthonormal basis ${\mathcal {B}}_k$. Then ${\mathbb {P}}_n(x_n,t_n;\ldots ;x_1,t_1)$ defines the joint probability of obtaining $\{x_n,\ldots ,x_1\}$ probing the system at times $\{t_n,\ldots ,t_1\}$ w.r.t. with (arbitrary) orthonormal bases $\{{\mathcal {B}}_n,\ldots ,{\mathcal {B}}_1\}$.

### Past-future independence

We shall conclude the work with the following observation. Recently, Budini and collaborators^56–58^ introduced an interesting non-Markovianity witness based on the so-called *conditional past-future* (CPF) correlations. Essentially, the vanishing of CPF correlations is equivalent to the validity of the regression equality (10) for projective measurements w.r.t. arbitrary orthonormal basis $\{{\mathcal {B}}_n,\ldots ,{\mathcal {B}}_1\}$ (cf. Remark 5.1).

In this regard, consider a normalized state vector

101 $$\begin{aligned} |{\widetilde{\Psi }}^{x_n,\ldots ,x_1}_{t_n,\ldots ,t_1}\rangle := \frac{| \Psi ^{x_n,\ldots ,x_1}_{t_n,\ldots ,t_1} \rangle }{\sqrt{{\mathbb {P}}_n(x_n,t_n;\ldots ;x_1,t_1)}} = |x_n\rangle \otimes |\varphi ^{x_n,\ldots ,x_1}_{t_n,\ldots ,t_1}\rangle , \end{aligned}$$

with $|\varphi ^{x_n,\ldots ,x_1}_{t_n,\ldots ,t_1}\rangle$ corresponding to the boson state vector which depends on the history of all *n* measurements up to time $t_n$. Now, the reduced dynamics starting at $t=t_n$

102 $$\begin{aligned} |x_n \rangle \!\langle x_n| \rightarrow {\textrm{Tr}}_B \Big ( U_\tau |x_n\rangle \!\langle x_n| \otimes |\varphi ^{x_n,\ldots ,x_1}_{t_n,\ldots ,t_1}\rangle \!\langle \varphi ^{x_n,\ldots ,x_1}_{t_n,\ldots ,t_1}| U^\dagger _\tau \Big ) \end{aligned}$$

provides a dynamical map which depends on the boson state vector $|\varphi ^{x_n,\ldots ,x_1}_{t_n,\ldots ,t_1}\rangle$, and hence it depends upon the entire measurement history up to time $t_n$. The process is Markovian whenever the above dynamical map *does not depend* upon the history of measurements records. For the multilevel spin–boson model we are considering, one has

103 $$\begin{aligned} {\textrm{Tr}}_B \Big ( U_\tau |x_n\rangle \!\langle x_n| \otimes |\varphi ^{x_n,\ldots ,x_1}_{t_n,\ldots ,t_1}\rangle \!\langle \varphi ^{x_n,\ldots ,x_1}_{t_n,\ldots ,t_1}| U^\dagger _\tau \Big ) = {\textrm{Tr}}_B \Big ( U_\tau |x_n\rangle \!\langle x_n| \otimes |{\textrm vac}\rangle \!\langle {\textrm vac}| U_\tau ^\dagger \Big ) = \Lambda _\tau ( |x_n\rangle \!\langle x_n|) , \end{aligned}$$

that is, one may erase the memory about measurement records replacing $|\varphi ^{x_n,\ldots ,x_1}_{t_n,\ldots ,t_1}\rangle$ by the vacuum state $|{\textrm vac}\rangle$.

## Conclusions

We have provided a detailed analysis of Markovianity and classicality for the multitime statistics associated with a paradigmatic model describing a multilevel system coupled with a multimode boson field, for which calculations can carried out explicitly, probed in an arbitrary fixed basis at different times. In the limit in which the coupling between the system and the environment is described by flat functions—that is, all field frequencies are coupled with the same strength—this system was proven to be Markovian for any measurement basis and any choice of the initial (pure) system–bath state. Furthermore, in the same limit, the model was proven to be classical if and only if the selected measurement basis coincides with the one (if any) that diagonalizes the reduced evolution of the excited sector; any other choice of basis will involve probability exchange between states competing with different eigenvectors, hence reveling the underlying quantum nature of the system. This result was compared with the characterization of classicality for quantum Markov processes in terms of the generation of coherence in the process, provided in^3,4^.

Our work provides a detailed exposition of fundamental concepts of growing interest in recent years—Markovianity and classicality—applied to particular instances of quantum systems having a relatively simple structure, while nevertheless retaining a sufficiently rich phenomenology of possible cases. In the context of our proposed model, the slightly counterintuitive fact that quantum systems *can* behave classically, when probed in a proper way, is easily interpreted in terms of the presence or absence of mixing between eigenvectors.

Among many possible future developments of our work, it would be useful to provide a similar discussion of the classicality of our model beyond the flat-coupling limit—that is, outside the Markovian scenario. In this case, classicality can be characterized in terms of the generation of quantum discord rather than coherence^4^. Intuitively, it would be tempting to conjecture that the model under investigation in the present work will behave classically when probed via the same measurement basis under which it is classical in the Markov regime: indeed, the reduced dynamics in the excited sector will be again diagonal in such a basis. We will leave a detailed answer of this question to future works. Finally, as a natural continuation of this line of research, *quantifying* nonclassicality in spin–boson models and their generalizations, thus going forward the mere characterization of processes that happen to be classical, would be of paramount importance. In particular, the classicality of both Markovian and non-Markovian dephasing processes deserves the thorough analysis (the classicality of particular models of such processes were recently analyzed in^59–63^).

## Supplementary Information

Supplementary Information.

## Data Availability

All data generated or analysed during this study are included in this published article and its supplementary information file.
