# Supplementary material for: On Markovianity and classicality in multilevel spin–boson models
Source: Sci Rep. 2023 Jan 27;13:1518. doi: 10.1038/s41598-023-28606-z (PMC9883298; doi:10.1038/s41598-023-28606-z)
Supplement: Supplementary file 1 — Supplementary Information. [file 41598_2023_28606_MOESM1_ESM.pdf]

Supplementary Information for On Markovianity and classicality in multilevel spin–boson models

Dariusz Chruściński[1,*], Samaneh Hesabi[1,†], and Davide Lonigro[2,3,‡]

[1]Institute of Physics, Faculty of Physics, Astronomy and Informatics, Nicolaus Copernicus University, Grudziadzka 5/7, 87-100 Toruń, Poland
[2]Dipartimento di Fisica and MECENAS, Università di Bari, I-70126 Bari, Italy
[3]INFN, Sezione di Bari, I-70126 Bari, Italy

A Proof of Prop. 3.1

Following [36] we prove the following result: given $\eta(\omega)$ let us consider the corresponding Fourier (anti–)transform

$$\hat{\eta}(x) := \int \mathrm{d}\omega \ \mathrm{e}^{\mathrm{i}\omega x}\eta(\omega). \tag{A.1}$$

Lemma A.1. *Let $\hat{\eta}(x) = 0$ for $x \in [-T, 0]$ (with $T > 0$). Then*

$$\mathrm{e}^{-\mathrm{i}t\boldsymbol{H}}\mathbb{1} \otimes b^\dagger_\alpha(\eta)\mathrm{e}^{\mathrm{i}t\boldsymbol{H}} = \mathbb{1} \otimes b^\dagger_\alpha\left(\mathrm{e}^{-\mathrm{i}t\omega}\eta\right) \qquad \forall t \in [0, T], \tag{A.2}$$

or equivalently, in the position representation,

$$\mathrm{e}^{-\mathrm{i}t\boldsymbol{H}}\mathbb{1} \otimes b^\dagger_\alpha(\hat{\eta})\mathrm{e}^{\mathrm{i}t\boldsymbol{H}} = \mathbb{1} \otimes b^\dagger_\alpha\left(\hat{\eta}(\cdot - t)\right) \qquad \forall t \in [0, T]. \tag{A.3}$$

Proof. Let us define

$$B^\dagger_\alpha(\eta; t) := \mathrm{e}^{-\mathrm{i}t\mathbf{H}}\mathbb{1}_S \otimes b^\dagger_\alpha(\eta)\mathrm{e}^{\mathrm{i}t\mathbf{H}}, \tag{A.4}$$

and note that this is the unique solution of the Heisenberg equation

$$\begin{cases} \frac{\mathrm{d}}{\mathrm{d}t}B^\dagger_\alpha(\eta; t) = -\mathrm{i}\left[\mathbf{H}, B^\dagger_\alpha(\eta; t)\right], \\ B^\dagger_\alpha(\eta; 0) = \mathbb{1} \otimes b^\dagger_\alpha(\eta). \end{cases} \tag{A.5}$$

We shall verify that

$$t \mapsto \mathbb{1} \otimes b^\dagger_\alpha(\mathrm{e}^{-\mathrm{i}t\omega}\eta) \tag{A.6}$$

solves Eq. (A.5) provided $\hat{\eta}(x) = 0$ for $x \in [-T, 0]$. Indeed, we have

$$\begin{aligned} \frac{\mathrm{d}}{\mathrm{d}t}\left(\mathbb{1}_S \otimes b^\dagger_\alpha(\mathrm{e}^{-\mathrm{i}t\omega}\eta)\right) &= \mathbb{1}_S \otimes \frac{\mathrm{d}}{\mathrm{d}t}b^\dagger_\alpha(\mathrm{e}^{-\mathrm{i}t\omega}\eta) \\ &= -\mathrm{i}\mathbb{1}_S \otimes [H_\mathrm{B}, b^\dagger_\alpha(\mathrm{e}^{-\mathrm{i}t\omega}\eta)], \end{aligned} \tag{A.7}$$

*darch@fizyka.umk.pl
†samaneh.hesabi@umk.pl
‡davide.lonigro@ba.infn.it

whereas

$$[\mathbf{H}, \mathbb{1} \otimes b_\alpha^\dagger(\mathrm{e}^{-\mathrm{i}t\omega}\eta)] = \mathbb{1} \otimes [H_\mathrm{B}, b_\alpha^\dagger(\mathrm{e}^{-\mathrm{i}\omega t}\eta)] + |\mathrm{e}_\alpha\rangle\langle\mathrm{g}| \otimes [b_\alpha(f_\alpha), b_\alpha^\dagger(\mathrm{e}^{-\mathrm{i}\omega t}\eta)]. \tag{A.8}$$

But

$$\begin{aligned}[b_\alpha(f_\alpha), b_\alpha^\dagger(\mathrm{e}^{-\mathrm{i}\omega t}\eta)] &= \int \mathrm{d}\omega\ f_\alpha(\omega)^* \mathrm{e}^{-\mathrm{i}\omega t}\eta(\omega) \\ &= \sqrt{\gamma_\alpha} \int \mathrm{d}\omega\ \mathrm{e}^{-\mathrm{i}\omega t}\eta(\omega) \\ &= \sqrt{\gamma_\alpha}\, \hat{\eta}(-t). \end{aligned} \tag{A.9}$$

Therefore, under our assumptions, the desired equality holds for all times $t \in [0, T]$. In particular, if $\eta(x) = 0$ for all $x \leq 0$, the desired equality holds for all times $t \geq 0$. □

B Proof of Prop. 4.3

Let us consider the particular case in which $[\mathrm{H_e}, \Gamma] = 0$, that is, both $\mathrm{H_e}$ and Γ are diagonal in the basis $\{|\mathrm{e}_x\rangle\}_{x=1,\dots,d}$. In this case, $\mathrm{A}(t)$ is diagonal as well in this basis, that is, by Eq. (43), choosing $|x\rangle = |\mathrm{e}_x\rangle$ for all $x = 1, \dots, d$, the 1-time probabilities become

$$\mathbb{P}_1(x, t) = \mathrm{e}^{-\gamma_x t} |\langle x|\psi_\mathrm{e}\rangle|^2; \tag{B.1}$$

$$\mathbb{P}_1(0, t) = 1 - \sum_{x=1}^{d} \mathrm{e}^{-\gamma_x t} |\langle x|\psi_\mathrm{e}\rangle|^2, \tag{B.2}$$

and the transition probabilities (80)–(81) simplify as

$$\mathbb{P}(x, t|y, s) = \mathrm{e}^{-\gamma_x(t-s)}\, \delta_{xy}, \tag{B.3}$$

$$\mathbb{P}(0, t|y, s) = 1 - \mathrm{e}^{-\gamma_y(t-s)}\,, \tag{B.4}$$

and all Chapman–Kolmogorov equations are easily satisfied. If, instead, $\{|x\rangle\}_{x=1,\dots,d}$ is a generic orthonormal basis of $\mathcal{H}_\mathrm{e}$, then, again by Eq. (43),

$$|\langle x|\mathrm{A}(t-s)|y\rangle|^2 = \sum_{j=1}^{d} \mathrm{e}^{-\gamma_j(t-s)} |\langle x|\mathrm{e}_j\rangle|^2\, |\langle y|\mathrm{e}_j\rangle|^2, \tag{B.5}$$

whence the transition probabilities (80)–(81) read

$$\mathbb{P}(x, t|y, s) = \sum_{j=1}^{d} \mathrm{e}^{-\gamma_j(t-s)} |\langle x|\mathrm{e}_j\rangle|^2\, |\langle y|\mathrm{e}_j\rangle|^2, \tag{B.6}$$

$$\begin{aligned}\mathbb{P}(0, t|y, s) &= 1 - \sum_{x=1}^{d}\sum_{j=1}^{d} \mathrm{e}^{-\gamma_j(t-s)} |\langle x|\mathrm{e}_j\rangle|^2\, |\langle y|\mathrm{e}_j\rangle|^2 \\ &= 1 - \sum_{j=1}^{d} \mathrm{e}^{-\gamma_j(t-s)} |\langle y|\mathrm{e}_j\rangle|^2\,, \end{aligned} \tag{B.7}$$

where we used the property $\sum_j |\langle x|\mathrm{e}_j\rangle|^2 = \||x\rangle\|^2 = 1$. Let us now consider the Chapman–Kolmogorov equality with $x = y$, that is, for all $x = 1,\dots,d$ and all $t \geq r \geq s \geq 0$,

$$\begin{aligned} 0 &= \mathbb{P}(x,t|x,s) - \sum_{z=0}^{d} \mathbb{P}(x,t|z,r)\mathbb{P}(z,r|x,s) \\ &= \sum_{j,\ell=1}^{d} \mathrm{e}^{-\gamma_j(t-r)}\mathrm{e}^{-\gamma_\ell(r-s)} |\langle x|\mathrm{e}_j\rangle|^2 |\langle x|\mathrm{e}_\ell\rangle|^2 \left[\delta_{j\ell} - \sum_{z=1}^{d} |\langle z|\mathrm{e}_j\rangle|^2 |\langle z|\mathrm{e}_\ell\rangle|^2\right]; \end{aligned} \tag{B.8}$$

that is, defining $\tau := t-s$ and $\sigma := r-s$, we must have

$$\forall\tau,\sigma\geq 0,\ \forall x = 1,\dots,d,\quad \sum_{j=1}^{d} \mathrm{e}^{-\gamma_j\tau} |\langle x|\mathrm{e}_j\rangle|^2 \sum_{\ell=1}^{d} \mathrm{e}^{-\gamma_\ell\sigma} |\langle x|\mathrm{e}_\ell\rangle|^2 \left[\delta_{j\ell} - \sum_{z=1}^{d} |\langle z|\mathrm{e}_j\rangle|^2 |\langle z|\mathrm{e}_\ell\rangle|^2\right] = 0; \tag{B.9}$$

but, since all γ_js are distinct and the exponential functions are linearly independent, the equality above holds if and only if

$$\forall x, j = 1,\dots,d,\quad \langle x|\mathrm{e}_j\rangle = 0 \ \text{ or } \ \forall\sigma\geq 0,\ \sum_{\ell=1}^{d} \mathrm{e}^{-\gamma_\ell\sigma} |\langle x|\mathrm{e}_\ell\rangle|^2 \left[\delta_{j\ell} - \sum_{z=1}^{d} |\langle z|\mathrm{e}_j\rangle|^2 |\langle z|\mathrm{e}_\ell\rangle|^2\right] = 0, \tag{B.10}$$

which, for the same reason, is in turn equivalent to

$$\forall x, j, \ell = 1,\dots,d,\quad \langle x|\mathrm{e}_j\rangle = 0 \ \text{ or } \ \langle x|\mathrm{e}_\ell\rangle = 0 \ \text{ or } \ \sum_{z=1}^{d} |\langle z|\mathrm{e}_j\rangle|^2 |\langle z|\mathrm{e}_\ell\rangle|^2 = \delta_{j\ell} \tag{B.11}$$

and, in particular,

$$\forall x, j = 1,\dots,d,\quad \langle x|\mathrm{e}_j\rangle = 0 \ \text{ or } \ \sum_{z=1}^{d} |\langle z|\mathrm{e}_j\rangle|^4 = 1. \tag{B.12}$$

Clearly, given $j = 1,\dots,d$, there must exist some $x = 1,\dots,d$ such that $\langle x|\mathrm{e}_j\rangle \neq 0$. Consequently,

$$\sum_{z=1}^{d} |\langle z|\mathrm{e}_j\rangle|^4 = 1. \tag{B.13}$$

Now, notice that $\sum_{z=1}^{d} |\langle z|\mathrm{e}_j\rangle|^2 = \||\mathrm{e}_j\rangle\|^2 = 1$. Consequently, the only case in which the equality above can hold is when all terms $|\langle z|\mathrm{e}_j\rangle|^2$ vanish expect a single one equaling one—that is, when the two bases coincide up to phase shifts; henceforth the claim.

C Proof of Theorem 4.2

Consider a projective measurements w.r.t. to $|x_\alpha\rangle$ ($\alpha = 0,1,\dots,d$) such that

$$|x_\alpha\rangle = \mu_\alpha|0\rangle \oplus |\tilde{x}_\alpha\rangle, \quad |\tilde{x}_\alpha\rangle \in \mathcal{H}_\mathrm{e}, \tag{C.1}$$

and $|\mu_\alpha|^2 + \||\tilde{x}_\alpha\rangle\|^2 = 1$. Taking $|\Psi_0\rangle = |\psi_0\rangle \otimes |\mathrm{vac}\rangle$, with $|\psi_0\rangle = \alpha|0\rangle \oplus |\psi_\mathrm{e}\rangle$ and using

$$U_{t_1}|\Psi_0\rangle = \Big[\alpha|0\rangle \otimes |\mathrm{vac}\rangle + \mathrm{A}(t_1)|\psi_\mathrm{e}\rangle\Big] \otimes |\mathrm{vac}\rangle + |0\rangle \otimes \sum_{j=1}^{d} b_j^\dagger(\xi_j(t_1))|\mathrm{vac}\rangle, \tag{C.2}$$

(see Section 3 of the main text), one finds

$$|x_1\rangle\langle x_1| \otimes \mathbb{1}_B U_{t_1}|\Psi_0\rangle = |x_1\rangle \otimes \left(\Big[\alpha\mu_1^* + \langle\psi_e|\mathrm{A}(t_1)|\psi_e\rangle\Big]|\mathrm{vac}\rangle + \mu_1^* \sum_{j=1}^{d} b_j^\dagger(\xi_j(t_1))|\mathrm{vac}\rangle \right). \quad \text{(C.3)}$$

Note that $|\Psi_{t_1}^{x_1}\rangle$ belongs now to the 2-excitation sector. Using the following property:

$$U_{\Delta t_2}\mathbb{1}_S \otimes b_j^\dagger(\xi_j(t_1))U_{\Delta t_2}^\dagger = \mathbb{1}_S \otimes b_j^\dagger(\xi_j(\Delta t_2, t_1)), \quad \text{(C.4)}$$

one easily proves the following equalities:

$$U_{\Delta t_2}|0\rangle \otimes |\mathrm{vac}\rangle = |0\rangle \otimes |\mathrm{vac}\rangle, \quad \text{(C.5)}$$

$$U_{\Delta t_2}|\tilde{x}_1\rangle \otimes |\mathrm{vac}\rangle = \mathrm{A}(\Delta t_2)|\tilde{x}_1\rangle \otimes |\mathrm{vac}\rangle + |0\rangle \otimes \sum_{k=1}^{d} b_k^\dagger(\tilde{\eta}_k^{(1)}(\Delta t_2))|\mathrm{vac}\rangle, \quad \text{(C.6)}$$

$$U_{\Delta t_2}|0\rangle \otimes b_j^\dagger(\xi_l(t_1))|\mathrm{vac}\rangle = |0\rangle \otimes b_j^\dagger(\xi_j(\Delta t_2, t_1))|\mathrm{vac}\rangle, \quad \text{(C.7)}$$

$$\begin{aligned} U_{\Delta t_2}|\tilde{x}_1\rangle \otimes b_j^\dagger(\xi_j(t_1))|\mathrm{vac}\rangle &= \mathrm{A}(\Delta t_2)|\tilde{x}_1\rangle \otimes b_j^\dagger(\xi_j(\Delta t_2, t_1))|\mathrm{vac}\rangle \\ &+ |0\rangle \otimes b_j^\dagger(\xi_j(\Delta t_2, t_1)) \sum_{k=1}^{d} b_k^\dagger(\tilde{\eta}_k^{(1)}(\Delta t_2))|\mathrm{vac}\rangle. \quad \text{(C.8)} \end{aligned}$$

Lemma C.1. *The following property holds:*

$$\left\| b_j^\dagger(\xi_j(\Delta t_2, t_1) \sum_{k=1}^{d} b_k^\dagger(\tilde{\eta}_k^{(1)}(\Delta t_2))|\mathrm{vac}\rangle \right\|^2 = \|\xi_j(t_1)\|^2 \sum_{k=1}^{d} \left\| \tilde{\eta}_k^{(1)}(\Delta t_2) \right\|^2. \quad \text{(C.9)}$$

Proof. One has

$$\left\| U_{\Delta t_2}|\tilde{x}_1\rangle \otimes b_j^\dagger(\xi_j(t_1))|\mathrm{vac}\rangle \right\| = \left\| |\tilde{x}_1\rangle \otimes b_j^\dagger(\xi_j(t_1))|\mathrm{vac}\rangle \right\|, \quad \text{(C.10)}$$

and hence

$$\begin{aligned} \left\| |\tilde{x}_1\rangle \otimes b_j^\dagger(\xi_j(t_1))|\mathrm{vac}\rangle \right\|^2 &= \Big\| \mathrm{A}(\Delta t_2)|\tilde{x}_1\rangle \otimes b_j^\dagger(\xi_j(\Delta t_2, t_1))|\mathrm{vac}\rangle \\ &\quad + |0\rangle \otimes b_j^\dagger(\xi_j(\Delta t_2, t_1)) \sum_{k=1}^{d} b_k^\dagger(\tilde{\eta}_k^{(1)}(\Delta t_2))|\mathrm{vac}\rangle \Big\|^2 \quad \text{(C.11)} \\ &= \left\| \mathrm{A}(\Delta t_2)|\tilde{x}_1\rangle \otimes b_j^\dagger(\xi_j(\Delta t_2, t_1))|\mathrm{vac}\rangle \right\|^2 \\ &+ \left\| b_j^\dagger(\xi_j(\Delta t_2, t_1)) \sum_{k=1}^{d} b_k^\dagger(\tilde{\eta}_k^{(1)}(\Delta t_2))|\mathrm{vac}\rangle \right\|^2. \quad \text{(C.12)} \end{aligned}$$

Finally, using the normalization condition

$$\|\mathrm{A}(\Delta t_2)|\tilde{x}_1\rangle\|^2 + \sum_{k=1}^{d} \|\tilde{\eta}_k^{(1)}(\Delta t_2))\|^2 = \||\tilde{x}_1\rangle\|^2, \quad \text{(C.13)}$$

one proves Eq. (C.9). □

Hence,

$$U_{\Delta t_2}\Psi_{t_1}^{x_1} = U_{\Delta t_2}|x_1\rangle \otimes \Big(\alpha_1(t_1)|\mathrm{vac}\rangle + \mu_1^* \sum_{j=1}^{d} b_j^\dagger(\xi_j(t_1))|\mathrm{vac}\rangle\Big), \tag{C.14}$$

with

$$\alpha_1(t_1) = \alpha\mu_1^* + \langle\tilde{x}_1|\mathrm{A}(t_1)|\psi_\mathrm{e}\rangle, \tag{C.15}$$

can be represented as follows:

$$\begin{aligned} U_{\Delta t_2}\Psi_{t_1}^{x_1} &= \mu_1|0\rangle \otimes \Big(\alpha_1(t_1)|\mathrm{vac}\rangle + \mu_1^* \sum_{j=1}^{d} b_j^\dagger(\xi_j(\Delta t_2, t_1))|\mathrm{vac}\rangle\Big) \\ &+ \alpha_1(t_1)\Big(\mathrm{A}(\Delta t_2)|\tilde{x}_1\rangle \otimes |\mathrm{vac}\rangle + |0\rangle \otimes \sum_{k=1}^{d} b_k^\dagger(\tilde{\eta}_k^{(1)}(\Delta t_2))|\mathrm{vac}\rangle\Big) \\ &+ \mu_1^*\Big(\mathrm{A}(\Delta t_2)|\tilde{x}_1\rangle \otimes b_j^\dagger(\xi_j(\Delta t_2, t_1))|\mathrm{vac}\rangle + |0\rangle \otimes b_j^\dagger(\xi_j(\Delta t_2, t_1)) \sum_{k=1}^{d} b_k^\dagger(\tilde{\eta}_k^{(1)}(\Delta t_2))|\mathrm{vac}\rangle\Big). \end{aligned} \tag{C.16}$$

Lemma C.2. *In the limit of flat form factors, then*

$$\langle\mathrm{vac}|b_j(\xi_j^*(\Delta t_2, t_1))b_k^\dagger(\tilde{\eta}_k^{(1)}(\Delta t_2))|\mathrm{vac}\rangle = 0, \tag{C.17}$$

for arbitrary $\Delta t_2 \geq 0$.

Proof. One has

$$\begin{aligned} &\langle\mathrm{vac}|b_j(\xi_j^*(\Delta t_2, t_1))b_k^\dagger\tilde{\eta}_k^{(1)}(\Delta t_2))|\mathrm{vac}\rangle \\ &= f_j^* f_k \int \mathrm{d}\omega \int_0^{t_1} \mathrm{d}s \int_0^{\Delta t_2} \mathrm{d}s' \mathrm{e}^{\mathrm{i}\omega\Delta t_2}\mathrm{e}^{\mathrm{i}\omega(t_1-s)}\mathrm{e}^{-\mathrm{i}\omega(\Delta t_2 - s')}\langle\psi_e|\mathrm{A}^\dagger(s)|\mathrm{e}_j\rangle\langle\mathrm{e}_k|\mathrm{A}(s')|\tilde{x}_1\rangle, \end{aligned} \tag{C.18}$$

and using

$$\int \mathrm{d}\omega\ \mathrm{e}^{\mathrm{i}\omega(t_1 - s + s')} = 2\pi\delta(s - [t_1 + s']),$$

which implies that $s = t_1 + s'$, whence s is outside the domain of integration $[0, t_1]$ thus implying Eq. (C.17). □

Finally, using the above lemma and the property (C.9), simple algebra leads to

$$\mathrm{Tr}_B U_{\Delta t_2}|\Psi_{t_1}^{x_1}\rangle\langle\Psi_{t_1}^{x_1}|U_{\Delta t_2}^\dagger = \Lambda_{\Delta t_2}(|x_1\rangle\langle x_1|)\mathbb{P}_1(x_1, t_1), \tag{C.19}$$

where

$$\mathbb{P}_1(x_1, t_1) = |\alpha\mu_1^* + \langle\tilde{x}_1|\mathrm{A}(t_1)|\psi_\mathrm{e}\rangle|^2 + |\mu_1|^2 \sum_{j=1}^{d} \|\xi_j(t_1)\|^2. \tag{C.20}$$

It should be clear from the above analysis that this scheme may be immediately generalized for n measurement scenario provided that Prop. 4.4 holds.

D Proof of Prop. 4.4

We shall prove it by induction: assuming that Eq. (99) in the main text holds for $(n-1)$ measurements, we will show that it holds for n measurements as well. One finds

$$\begin{aligned}
& U_{\Delta t_{n+1}} |\tilde{x}_{n+1}\rangle \otimes b^{\dagger}_{j_1}(\xi_{j_1}(t_n - t_1, \Delta t_1)) \prod_{k=2}^{n} \sum_{j_k=1}^{d} b^{\dagger}_{j_k}(\tilde{\eta}^{(k-1)}_{j_k}(t_n - t_k, \Delta t_k))|\mathrm{vac}\rangle \\
& = \mathrm{A}(\Delta t_{n+1})|\tilde{x}_{n+1}\rangle \otimes b^{\dagger}_{j_1}(\xi_{j_1}(t_{n+1} - t_1, \Delta t_1)) \prod_{k=2}^{n} \sum_{j_k=1}^{d} b^{\dagger}_{j_k}(\tilde{\eta}^{(k-1)}_{j_k}(t_{n+1} - t_k, \Delta t_k))|\mathrm{vac}\rangle \\
& + |0\rangle \otimes b^{\dagger}_{j_1}(\xi_{j_1}(t_{n+1} - t_1, \Delta t_1)) \prod_{k=2}^{n+1} \sum_{j_k=1}^{d} b^{\dagger}_{j_k}(\tilde{\eta}^{(k-1)}_{j_k}(t_{n+1} - t_k, \Delta t_k))|\mathrm{vac}\rangle. \qquad \text{(D.1)}
\end{aligned}$$

Hence, due to the fact that $U_{\Delta t_{n+1}}$ is unitary,

$$\begin{aligned}
& \| \tilde{x}_{n+1}\rangle\|^2 \left\| b^{\dagger}_{j_1}(\xi_{j_1}(t_n - t_1, \Delta t_1)) \prod_{k=2}^{n} \sum_{j_k=1}^{d} b^{\dagger}_{j_k}(\tilde{\eta}^{(k-1)}_{j_k}(t_n - t_k, \Delta t_k))|\mathrm{vac}\rangle \right\|^2 \\
& = \|\mathrm{A}(\Delta t_{n+1})|\tilde{x}_{n+1}\rangle\|^2 \left\| b^{\dagger}_{j_1}(\xi_{j_1}(t_{n+1} - t_1, \Delta t_1)) \prod_{k=2}^{n} \sum_{j_k=1}^{d} b^{\dagger}_{j_k}(\tilde{\eta}^{(k-1)}_{j_k}(t_{n+1} - t_k, \Delta t_k))|\mathrm{vac}\rangle \right\|^2 \\
& + \left\| b^{\dagger}_{j_1}(\xi_{j_1}(t_{n+1} - t_1, \Delta t_1)) \prod_{k=2}^{n+1} \sum_{j_k=1}^{d} b^{\dagger}_{j_k}(\tilde{\eta}^{(k-1)}_{j_k}(t_{n+1} - t_k, \Delta t_k))|\mathrm{vac}\rangle \right\|^2 . \qquad \text{(D.2)}
\end{aligned}$$

Now, using Eq. (99), i.e.

$$\begin{aligned}
& \left\| b^{\dagger}_{j_1}(\xi_{j_1}(t_{n+1} - t_1, \Delta t_1)) \prod_{k=2}^{n} \sum_{j_k=1}^{d} b^{\dagger}_{j_k}(\tilde{\eta}^{(k-1)}_{j_k}(t_{n+1} - t_k, \Delta t_k))|\mathrm{vac}\rangle \right\|^2 \qquad \text{(D.3)} \\
& = \left\| b^{\dagger}_{j_1}(\xi_{j_1}(\Delta t_1)) \right\|^2 \prod_{k=2}^{n} \left\| \sum_{j_k=1}^{d} b^{\dagger}_{j_k}(\tilde{\eta}^{(k-1)}_{j_k}(t_{n+1} - t_k, \Delta t_k)) \right\|^2 ,
\end{aligned}$$

one obtains

$$\begin{aligned}
& \left\| b^{\dagger}_{j_1}(\xi_{j_1}(\Delta t_1)) \prod_{k=2}^{n+1} \sum_{j_k=1}^{d} b^{\dagger}_{j_k}(\tilde{\eta}^{(k-1)}_{j_k}(t_{n+1} - t_k, \Delta t_k))|\mathrm{vac}\rangle \right\|^2 \qquad \text{(D.4)} \\
& = \Big(\||\tilde{x}_{n+1}\rangle\|^2 - \|\mathrm{A}(\Delta t_{n+1}|\tilde{x}_{n+1}\rangle\|^2 \Big) \|b^{\dagger}_{j_1}(\xi_{j_1}(\Delta t_1))\|^2 \prod_{k=2}^{n} \left\| \sum_{j_k=1}^{d} b^{\dagger}_{j_k}(\tilde{\eta}^{(k-1)}_{j_k}(t_{n+1} - t_k, \Delta t_k)) \right\|^2 .
\end{aligned}$$

Finally, using the normalization property

$$\|\mathrm{A}(\Delta t_{n+1})|\tilde{x}_{n+1}\rangle\|^2 + \sum_{j=1}^{d} \left\| \tilde{\eta}^{(n)}_{j}(\Delta t_{n+1}) \right\|^2 = \||\tilde{x}_{n+1}\rangle\|^2, \qquad \text{(D.5)}$$

one proves

$$\left\| b^\dagger_{j_1}(\xi_{j_1}(\Delta t_1)) \prod_{k=2}^{n+1} \sum_{j_k=1}^{d} b^\dagger_{j_k}(\tilde{\eta}^{(k-1)}_{j_k}(t_{n+1}-t_k, \Delta t_k))|\mathrm{vac}\rangle \right\|^2$$

$$= \|\xi_{j_1}(\Delta t_1)\|^2 \prod_{k=2}^{n+1} \left\| \sum_{j_k=1}^{d} \tilde{\eta}^{(k-1)}_{j_k}(t_{n+1}-t_k, \Delta t_k) \right\|^2 . \tag{D.6}$$
